# *In Vivo* Assessment of Bone Regeneration in Alginate/Bone ECM Hydrogels with Incorporated Skeletal Stem Cells and Single Growth Factors

**DOI:** 10.1371/journal.pone.0145080

**Published:** 2015-12-16

**Authors:** David Gothard, Emma L. Smith, Janos M. Kanczler, Cameron R. Black, Julia A. Wells, Carol A. Roberts, Lisa J. White, Omar Qutachi, Heather Peto, Hassan Rashidi, Luis Rojo, Molly M. Stevens, Alicia J. El Haj, Felicity R. A. J. Rose, Kevin M. Shakesheff, Richard O. C. Oreffo

**Affiliations:** 1 Bone and Joint Research Group, Centre for Human Development, Stem Cells and Regeneration, Institute of Developmental Sciences, University of Southampton, Southampton, SO16 6YD, United Kingdom; 2 Wolfson Centre for Stem Cells, Tissue Engineering and Modelling, School of Pharmacy, University of Nottingham, Centre for Biomolecular Sciences, University Park, Nottingham, NG7 2RD, United Kingdom; 3 Locate Therapeutics Limited, MediCity, Nottingham, NG90 6BH, United Kingdom; 4 Department of Materials, Imperial College London, Royal School of Mines, London, SW7 2AZ, United Kingdom; 5 Department of Bioengineering, Imperial College London, South Kensington Campus, London, SW7 2AZ, United Kingdom; 6 Institute for Biomedical Engineering, Imperial College London, South Kensington Campus, London, SW7 2AZ, United Kingdom; 7 Biomaterials, Biomimetics, Biophotonics Research Division, King's College London, Dental Institute, Guy's Hospital, Tower Wing, London Bridge, London SE1 9RT, United Kingdom; 8 Institute for Science and Technology in Medicine, Keele University, Guy Hilton Research Centre, Stoke-on-Trent, ST4 7BQ, United Kingdom; University of California, San Diego, UNITED STATES

## Abstract

The current study has investigated the use of decellularised, demineralised bone extracellular matrix (ECM) hydrogel constructs for *in vivo* tissue mineralisation and bone formation. Stro-1-enriched human bone marrow stromal cells were incorporated together with select growth factors including VEGF, TGF-β3, BMP-2, PTHrP and VitD3, to augment bone formation, and mixed with alginate for structural support. Growth factors were delivered through fast (non-osteogenic factors) and slow (osteogenic factors) release PLGA microparticles. Constructs of 5 mm length were implanted *in vivo* for 28 days within mice. Dense tissue assessed by micro-CT correlated with histologically assessed mineralised bone formation in *all* constructs. Exogenous growth factor addition did not enhance bone formation further compared to alginate/bone ECM (ALG/ECM) hydrogels alone. UV irradiation reduced bone formation through degradation of intrinsic growth factors within the bone ECM component and possibly also ECM cross-linking. BMP-2 and VitD3 rescued osteogenic induction. ALG/ECM hydrogels appeared highly osteoinductive and delivery of angiogenic or chondrogenic growth factors led to altered bone formation. All constructs demonstrated extensive host tissue invasion and vascularisation aiding integration and implant longevity. The proposed hydrogel system functioned without the need for growth factor incorporation or an exogenous inducible cell source. Optimal growth factor concentrations and spatiotemporal release profiles require further assessment, as the bone ECM component may suffer batch variability between donor materials. In summary, ALG/ECM hydrogels provide a versatile biomaterial scaffold for utilisation within regenerative medicine which may be tailored, ultimately, to form the tissue of choice through incorporation of select growth factors.

## Introduction

There is a growing socio-economic need for new efficacious methods to repair bone damage *in vivo*. In an increasingly aged population, bone defects resulting from trauma [1, 2], disease (osteonecrosis [3], osteoporosis [4, 5], and Paget’s disease [6]), and/or surgical intervention (bone excision [7]), pose a significant challenge and accentuate the need for bone tissue engineering strategies [8]. Current clinical reparative practice involves the utilisation of autogenic and allogenic bone grafts for replacement, repair, and regeneration [9, 10]. However, limitations include availability, complex decellularisation and sterilisation procedures, disease transmission, donor site morbidity, bone union efficacy, immunogenic/inflammatory responses as well as significant variability in osteoconductivity, osteoinductivity, and vascularisation capacity. To address some of these issues, osteogenic growth factors such as bone morphogenetic protein 2 (BMP-2) can be combined with autografts and allografts to improve clinical outcome [11, 12]. Furthermore, rapid clearance, a need for localised concentration, and a short half-life *in vivo*, have all contributed to the requirement of supraphysiological (milligram) doses of BMPs to elicit a sufficient bone healing response. Consequently, a platform shift towards tissue-engineered constructs is a current research focus [13–15].

Bone tissue engineering has centred on a number of fundamental cornerstones; i) an inducible cell source, ii) inductive signalling, and iii) an appropriate, potentially bioactive scaffold. For bone bioengineering, skeletal stem cells (SSCs) provide an ideal cell source due to their self-renewal and osteochondral differentiation potential. Select growth factors can provide inductive signals for angio-, chondro-, and osteo-genesis whilst delivery vehicles can be utilised to encapsulate these growth factors and provide spatiotemporal release *in vivo*, recreating microenvironmental and developmental cues [16, 17]. Encapsulation can be achieved within the scaffold material or within microparticles added to the scaffold [18–21]. Finally, the scaffold component typically offers structural integrity and support during cellular colonisation and eventual tissue regeneration. Suitable scaffolds should support and direct cell growth and tissue formation, replicate native 3D architecture, withstand mechanical and physiological loading and stresses, and exhibit function-dependent biodegradation without production of toxic by-products [22–25].

Hydrogels have long been used for tissue regeneration due to their capacity to replicate characteristics inherent to extracellular matrix (ECM) [26–28]. Hydrogels can in part, mimic *in vivo* environments replicating cell-matrix, and enabling homo/hetero-geneous cell-cell interactions [29]. Furthermore, hydrogels can aid nutrient and gaseous transfer, removal of metabolic waste products and signal transduction, fulfilling their clinical application as a minimally invasive injectable (due to their liquid/gel structure) void filler for *in vivo* tissue regeneration [30]. However, there remains debate over the structure and composition of hydrogels and how these features affect clinical efficacy [31]. Hydrogels typically no longer resemble the intrinsic 3D architecture of native tissues, and it is unclear whether essential microenvironmental cues including cytokines, growth factors, and hormones are retained [29]. To address these concerns, hydrogels can be tailored to exhibit desired material properties [32] including i) charge and porosity [33–35], ii) structure and scaffold mechanics [36, 37] and iii) the addition of growth factors (i.e. BMP-2 for bone) to reconstitute in part, microenvironmental cues [38] to aid tissue regeneration [39–42].

The current study has examined a bone ECM-derived hydrogel [43–45] based on previous work using demineralised bone matrix (DBM), including application in an *ex vivo* organotypic model [46–49]. *In vivo* studies utilising DBM have long demonstrated capacity for bone regeneration [43–45]. However, use of DBM has resulted in variable clinical success due to study dependent differences in processing and preparation, donor variability, and inflammatory response [50, 51]. Derivation of purified bone ECM through removal of cell debris and lipids, previously described by Sawkins M. J. *et al* [52], has produced a hydrogel matrix composed primarily of macromolecules, highly conserved across animal species which reduces potential immunogenic and inflammatory response [53, 54]. This bone ECM component has been combined with alginate to improve structural stability through ionic cross-linking. The new alginate/bone ECM (ALG/ECM) hydrogels were seeded with SSCs (Stro-1-enriched human bone marrow stromal cells (HBMSCs)) and growth factor loaded microparticles to examine bone formation capacity. Microparticles were fabricated from a widely used biodegradable hydrophilic polymer, poly(D,L-lactic-co-glycolic acid) (P_DL_LGA) [55–58], combined with an ‘in-house’ Triblock polymer (P_DL_LGA-PEG-P_DL_LGA) [59, 60]. Microparticles were fabricated in two sizes with different volumes of Triblock, providing two distinct release profiles. Large (50–100μm) microparticles with 30% Triblock provided a fast release profile, whilst small (20–30μm) microparticles with 10% Triblock provided a slow release profile [60]. Microparticles were loaded with individual angiogenic (VEGF–fast release), chondrogenic (TGF-β3 –fast release), and osteogenic (BMP-2, PTHrP, or VitD3 –slow release) growth factors, and human serum albumin (HSA) as a carrier protein. Hydrogel constructs were implanted subcutaneously within immuno-deficient mice for 28 days to assess bone formation.

The current study has employed a multifactorial approach to bone tissue engineering; thus osteoinductive ECM hydrogels, stabilised with alginate, were loaded with SSCs and microparticle-encapsulated growth factors to investigate bone development and tissue formation *in vivo*. The long-term goal was to assess whether a multifaceted approach could improve on current bone regeneration strategies. Based on current knowledge in the bone bioengineering field, the authors hypothesise the proposed system combines elements from several tissue engineering approaches to enhance bone formation *in vivo*, comparable to that required in a clinical setting. Specifically, we hypothesise, ECM hydrogels containing growth factor loaded microparticles can induce bone formation.

## Materials and Methods

All materials were purchased from Sigma Aldrich unless otherwise stated.

### Ethics statement

The use of human material in this study was approved by the NHS/University of Southampton local research governing office (ref no: 6418) and by the Southampton NHS research and development committee (ref no: T&O/079) as well as the Southampton and South West Hampshire Local Research Ethics Committee (LREC: 194/99/1). Human bone marrow was collected following informed consent (approval from the LREC) from patients undergoing total hip-replacement surgery. Written consent was obtained from each informed patient prior to surgery. Information was recorded and logged on a central database. The consent form was maintained with patients’ medical-records files. According to the terms of the ethical approval, the patient samples were anonymised/unlinked. We received only basic information including the age and gender of each patient sample. Collection of samples was recorded and reported annually to the ethical research governing body and the Human Tissue Authority as approved by the Southampton and South West Hampshire LREC. All animal procedures were carried out in accordance with the guidelines and regulations laid down in the Animals (Scientific Procedures) Act 1986. MF1 nu/nu mice were sacrificed after 28 days by schedule 1 CO_2_ inhalation and cervical dislocation according to Home Office Approval UK (Project license–PPL 30/2762). All surgery was performed under anaesthesia/analgesia, and all efforts were made to minimise suffering.

### Marrow preparation and cell isolation

Human bone marrow was collected from haematologically normal osteoarthritic patients (4 patients, mean age 63) undergoing total hip-replacement surgery at the Southampton General Hospital. Cells were isolated and enriched as described by Gothard D. *et al*. [61] In brief, marrow was suspended in modified eagle’s medium—alpha (α-MEM) and centrifuged at 1000 rpm for 4 min to remove fat. Marrow was then passed through a 40 μm sieve to remove blood clots and bone fragments. Red blood cells were also removed via centrifugation at 2200 rpm and 18°C for 40 min using LymphoPrep™ (Lonza). Isolated cells were washed with phosphate buffered saline (PBS, Lonza) and suspended in blocking buffer (α-MEM, 10% human serum, 5% foetal calf serum (FCS) and 10 mg/mL bovine serum albumin (BSA)) for 15 min at 4°C. Cells were then incubated with neat primary antibody (anti-human Stro-1+, Hybridoma) for 30 min at 4°C before washing with isolation buffer (0.5% BSA and 2 mM ethylenediaminetetraacetic acid (EDTA) in dH_2_O) as previously detailed [62]. Labelled cells were then incubated with magnetic bead conjugated secondary antibody (200 μL in 800 μL isolation buffer, Miltenyi Biotec) for 15 min at 4°C before further washes. Stro-1+ cells were then separated by MACS before *in vitro* culture expansion in basal media (α-MEM, 10% FCS, penicillin (100 U/mL) and streptomycin (0.1 mg/mL)) to 80% confluency. Cells were cultured at 37°C and 5% CO_2_ in a humidified atmosphere to P2 prior to hydrogel incorporation.

### Growth factor loaded microparticles

Poly (DL-lactide-co-glycolide) (P_DL_LGA, Lakeshore Biomaterials Inc., USA) microparticles were prepared using a water-in-oil-in-water (w/o/w) emulsion method as described previously [59, 60]. Growth factors were dissolved in 100 μL of 10% (w/v) aqueous human serum albumin (HSA) (120 μg human recombinant VEGF (PeproTech, UK), 40 μg human recombinant TGF-β3 (PeproTech, UK), 1 mg of human recombinant BMP-2 (in-house Hybridoma construct), or 0.8 mg PTHrP (PeproTech, UK)) and added to a solution of P_DL_LGA and P_DL_LGA-PEG (poly ethylene glycol)-P_DL_LGA Triblock in dichloromethane to form a primary water-in-oil emulsion through homogenisation at 9,000 rpm for 2 min using a Silverson L5M homogeniser (Silverson Machines, UK). A double emulsion was formed through homogenisation in 200 mL 0.3% (w/v) poly vinyl acetate (PVA) solution for 2 min at 9,000 rpm (HSA/BMP-2) or 2,000 rpm (HSA/VEGF, HSA/TGF-β3 and HSA/PTHrP). VitD3 loaded microparticles were fabricated using a single oil-in-water emulsion method by adding 25 μg of VitD3 to the polymer solution (P_DL_LGA with 10% P_DL_LGA-PEG-P_DL_LGA Triblock in dichloromethane) before homogenisation at 9,000 rpm for 2 min.

Resultant emulsions were magnetically stirred at 300 rpm for a minimum 4 h before filtration and lyophilisation of resultant microparticles. Two formulations were implemented for temporal release of loaded growth factors; fast (large microparticles (50–100 μm) formed with P_DL_LGA (85:15, 50 kDa) and 30% (w/w) Triblock copolymer) release profile for HSA/VEGF and HSA/TGF-β3, and slow (small microparticles (20–30 μm) formed with P_DL_LGA (50:50, 55 kDa) with 10% (w/w) Triblock copolymer) release profile for HSA/BMP-2, HSA/PTHrP, and HSA/VitD3. HSA and/or blank P_DL_LGA microparticles were used as controls for growth factors and VitD3 loaded particles, respectively [52].

Growth factors and loading concentrations were selected based on their previously investigated capacity to induce angio-, chondro- and osteo-genesis within SSC populations *in vitro* (unpublished data).

### Alginate and bone ECM preparation

Low viscosity alginate (Acros Organics, Fisher Scientific, UK) was prepared under sterile conditions as a 2% (w/v) solution in calcium free Dulbecco’s Modified Eagle’s Medium (DMEM, Invitrogen, UK) and pasteurised for 1 h at 65°C (conventional UV irradiation or autoclaving damaged cross-linking; 2% solution was too thick for filter sterilisation). Decellularised and demineralised bone ECM digest (10 mg/mL) was prepared as previously described by Sawkins M. J. 2013 [52]. Briefly, liquid nitrogen was used to freeze and fragment bovine cancellous bone, prior to demineralisation for 24 h at room temperature in 0.5 M HCl (25 mL/g bone). Chloroform/methanol solution (Fisher Scientific, UK) was used to remove lipids before washing with dH_2_O, snap freezing and lyophilisation.

Decellularisation was performed through agitation in 0.05% trypsin/0.02% EDTA at 37°C and 5% CO_2_ for 24 h before repeat snap freezing and lyophilisation. Prepared bone powder was subsequently combined with 1 mg/mL pepsin in 0.01 M HCl for a final concentration of 10 mg/mL, and stirred at room temperature for 96 h until the entire matrix was dissolved. Bone digests were finally aliquoted and stored at -20°C.

Two control hydrogels were prepared to investigate whether the bone ECM component retained bioactive growth factors; i) bone ECM was replaced with collagen (ALG/Col, Type I rat tail collagen), ii) bone ECM was irradiated under U.V. overnight (irradiated ALG/ECM). Collagen was used to replace bone ECM to assess whether effects were attributable to the bone ECM component. U.V. irradiation was performed to inactivate endogenous growth factors within bone ECM.

### Hydrogel preparation and *in vivo* implantation

Hydrogels were prepared as a 3:2 mixture (volume) of bone ECM and low viscosity alginate, respectively. Both growth factor loaded microparticles and human Stro-1+ enriched cells (1 x 10^6^ cells/mL) were incorporated within the hydrogels during formation. 5 mg of appropriate microparticles were suspended in 5 μL calcium free DMEM and added to each hydrogel so that final concentrations of bioactive growth factors were 50 ng/mL (VEGF), 15 ng/mL (TGF-β3), 100 ng/mL (BMP2), 100 ng/mL (PTHrP), and 25 nM (VitD3) (based on previous work describing minimum concentration required to elicit an effect on cell cultures). 1.5 mL hydrogel was prepared with 0.4 mL alginate (vortexed with microparticles), 0.6 mL bone ECM digest and 0.5 mL calcium free DMEM (with or without Stro-1+ cells) and mixed thoroughly between two syringes attached by a luer lock (NHS Supplies, UK). Afterwards, the mixture was pipetted into 135 mM calcium chloride for 10 min to allow cross-linking, forming long thin cylindrical structures. Set hydrogel structures were then incubated in culture media (α-MEM, penicillin (100 U/mL), streptomycin (0.1 mg/mL) and L-ascorbic acid 2-phosphate (100 μM)) supplemented with 2.7 mM calcium chloride overnight at 37°C in 5% CO_2_ and a humidified atmosphere, to avoid introducing the initial burst release of growth factors from the microparticle surface *in vivo* (based on a washing step for the microparticles previously published by Smith E. L. *et al* [48, 49]). 5 mm length segments were measured and cut from each hydrogel in preparation for subcutaneous implantation *in vivo* within MF1 nu/nu immuno-deficient mice bilaterally along the back (3 implants separately spaced per group per side; n = 3). One side received hydrogel implants with Stro-1+ cell incorporation, whilst the other side received hydrogel implants without Stro-1+ cell incorporation. Each mouse received one growth factor loaded hydrogel group. Implants were harvested after 28 days and fixed in 4% paraformaldehyde (PFA). Both BMP-2 and VitD3 groups were repeated following promising bone formation over other growth factors (n = 6, in total).

### Micro-computed tomography

Quantitative 3D analysis of hydrogels was performed using a SkyScan 1176 scanning system (Bruker micro-CT, Kontich). Samples were scanned at 18 μm resolution and reconstructed using NRecon software interface (v.1.6.4.6, Bruker micro-CT, Kontich). Reconstructed hydrogels were analysed using CT Analyser (v.1.13.2.1+, Bruker micro-CT, Kontich) to assess tissue volume (TV), percentage bone volume (PBV), bone surface to bone volume ratio (BS/BV), trabecular number (Tb.N), trabecular thickness (Tb.T), and trabecular separation (Tb.S). Bone and soft tissue greyscale values were assessed according to standard phantom scans, and intact mouse bone (forearm) scans. Bone tissue was assessed using greyscale values 80–255 (S1A Fig), as wider values (60–255) began to highlight soft tissue (S1B Fig). Soft tissue analysis was performed using greyscale values 20–255.

### Histology

Following micro-CT analysis, samples were imaged for macroscopic differences using a PowerShot G10 camera (Canon) mounted on a stereomicroscope (Leica, UK). Samples were then dehydrated through a series of ethanol washes (50%, 90% and 100% in dH_2_O) and incubation within Histo-Clear (National Diagnostics, UK). Following incubation in paraffin wax for 1 h at 60°C, samples were embedded in wax blocks using an automated Shandon Citadel 2000. Consecutive 7 μm thick sections were rehydrated through Histo-Clear, graded ethanol and dH_2_O before staining with Alcian blue/Sirius red (A/S), Von Kossa (VK), and Goldner’s Trichrome (GT). A/S involved staining with Weigert’s haematoxylin, 0.5% Alcian blue (stains proteoglycan-rich cartilage matrix), and 1% Sirius red (stains collagenous matrix). VK involved staining with silver nitrate (under U.V.), sodium thiosulfate, Alcian blue, and van Gieson. GT involved staining with Weigert’s haematoxylin, ponceau-fuchsin-azophloxin, phosphomolybdic acid, and light green. Sections were then dehydrated and mounted with DPX before imaging on an Olympus BX-51/22 dotSlide digital virtual microscope using OlyVIA 2.1 software (Olympus Soft Imaging Solutions, GmBH, UK). Duplicate sections per sample were processed for each stain (n = 3 to 6 samples).Additional sections were treated with hydrogen peroxide (3% in dH2O) to quench endogenous peroxidase activity. Sections were incubated with blocking buffer (1% BSA in PBS) for 15 min then primary antibody solution (anti-von Willebrand factor (vWF) or anti-vimentin) overnight at 4°C. Biotinylated secondary antibody (1:200 in blocking buffer) was applied for 1 h prior to incubation with avidin-conjugated peroxidase. Sections were treated with 3-amino-9-ethylcarbazole for 10 min or until a red-brown reaction product could be visualised. Stained sections were subsequently washed and counterstained with haematoxylin.

### Histomorphometry

Quantification of growth parameters including tissue invasion, matrix deposition, mineralisation, and vascularisation was performed through colour threshold analysis of scanned images using an optimised macro in Fiji (Image J) [63]. Initially a ‘region of interest’ (ROI) was drawn around the total area of each hydrogel sample before further ROIs were created highlighting missing or torn areas of hydrogel, and both selections were saved as a ROI file. Automated analysis using the macro was then performed on all images using corresponding ROI files (S2A Fig). The macro functioned through ‘colour threshold’ selection (hue, saturation and brightness values; 0–255) and creation of a black (selected colour) and white (non-selected colour) mask. A point grid was then overlaid on the masks where each point constituted 5 by 5 pixels; approximate resolution of a single cell. Saved ROIs were then applied to define areas for quantification. Both positive points (≥ 50% black pixels) and total number of points within the sample area were counted and used to calculate the selected colour as a percentage of the total sample. Points corresponding to torn ROIs were added where appropriate.

A/S–blue (hue 120–150, saturation 50–255, brightness 0–255) represented proteoglycans and residual hydrogel, red (hue 210–255, saturation 20–255, brightness 0–255) represented tissue invasion, and purple (hue 140–200, saturation 50–255, brightness 140–255) represented collagen deposition (S2B Fig).

VK—black (hue 0–255, saturation 0–255, brightness 0–100) represented mineralisation, and pink (hue 170–255, saturation 100–255, brightness 50–255) represented cell invasion (S2C Fig).

GT—green represented osteoid, purple represented cell invasion, and red represented erythrocytes (S2D Fig). Quantification of erythrocytes (identified by colour and morphology) assessed vascularisation of the hydrogel constructs. A square grid of known size (200 μm) was overlaid on each image and the number of squares containing bright red erythrocytes were counted and calculated as a percentage of the total number of squares covering the area of the hydrogel. Macro quantification could not be used to assess red staining due to low occurrence and surface area.

All hydrogel implants exhibited areas of dense stain which were torn and shredded. These areas were quantified (S3Ai and S3Bi Fig) and added to the total area of the immediate surrounding colour (blue in A/S red stains, and black in VK stains). Similar statistical results were observed compared to datasets without torn area incorporation (S3Aii, S3Bii, S9A and S10A Figs).

### Statistical analysis

All data sets are presented as mean +/- standard deviation. Statistical analysis was performed using GraphPad InStat3 v3.06 software. Differences between experimental groups were determined for statistical significance by use of a one-way ANOVA with Tukeys multiple comparison test. Intra-group differences between those with and without Stro-1+ cells were assessed by a Welch corrected unpaired t-test. Correlation between datasets was assessed using a linear regression model (Pearson). ‡ denotes positive correlation. Emboldened columns denote intra-group significance. Significance is depicted as * P ≤ 0.05, ** P ≤ 0.01, *** P ≤ 0.001.

## Results

### Hydrogel mineralisation and bone formation assessed by micro CT analysis

**Fig 1:**
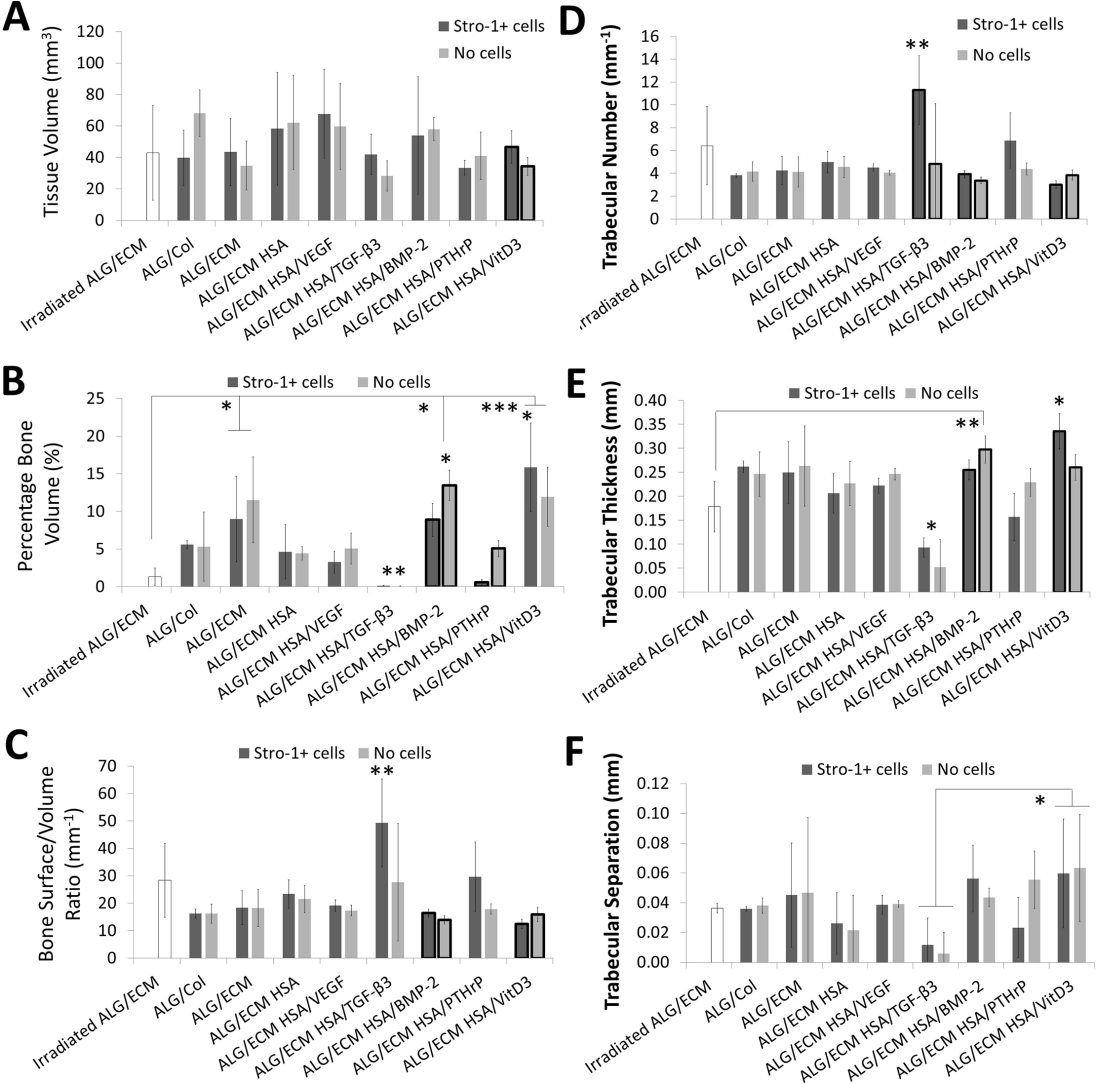
Micro CT analysis of hydrogels following 28 days *in vivo* implantation. Tissue volume (**A**), percentage bone volume (**B**), bone surface to volume ratio (**C**), trabecular number (**D**), trabecular thickness (**E**) and trabecular separation (**F**) were all assessed between growth factor groups. Emboldened columns depict statistically significant intragroup differences between those with and without Stro-1+ cell incorporation. Asterisks depict statistical difference between the group above which the asterisk is positioned and all the other groups; if positioned centrally above both groups with and without Stro-1+ cell incorporation, statistical difference was observed for both compared across all groups. Error bars are S.D. * P ≤ 0.05, ** P ≤ 0.01, *** P ≤ 0.001.

Following harvest, after 28 days implantation, hydrogel samples were imaged by stereomicroscopy (S4 Fig) prior to micro CT assessment of bone formation (Fig 1). There were no major differences in the appearance of hydrogels between growth factor groups or between samples with and without Stro-1+ cell incorporation. All samples exhibited similar blood vessel and tissue invasion upon examination and micro CT analysis showed no significant differences in tissue volume (TV) between any two individual hydrogel groups (Fig 1A). Large opaque white areas could be seen in most samples which were rigid upon manipulation and therefore potential areas of mineralisation. Minimal or no opaque areas were observed within HSA/TGF-β3 hydrogels, supported by negligible bone tissue formation evidenced through micro CT analysis (Fig 1B).

No groups were significantly different compared to control ALG/ECM for any parameter assessed by micro CT. However, significant differences were observed in comparison to control irradiated ALG/ECM, including significantly (P ≤ 0.05) greater bone formation observed within both HSA/BMP-2 and HSA/VitD3 groups (Fig 1B). HSA/VitD3 exhibited significantly higher trabecular thickness (Tb.T) (P ≤0.05) compared to all other groups except HSA/PTHrP (Fig 1E). ALG/ECM, BMP-2 and VitD3 demonstrated significantly (P ≤ 0.05) increased percentage bone volume (PBV) compared to control irradiated ALG/ECM (dependent on Stro-1+ cell incorporation) and chondrogenic factor TGF-β3. Indeed, ALG/ECM and HSA/BMP-2 *without* incorporation and HSA/VitD3 *with* incorporation exhibited significantly (P ≤ 0.05) enhanced bone formation. Interestingly, HSA/BMP-2 and HSA/VitD3 often exhibited significant (P ≤ 0.05) intra-group differences between those with and without Stro-1+ cells (S7 Fig). HSA/TGF-β3 and HSA/PTHrP were also shown to exhibit significant intra-group differences, dependent on bone parameter. HSA/TGF-β3 exhibited significantly (P ≤ 0.01) greater bone surface to bone volume ratio (BS/BV) and trabecular number (Tb.N), but lower (P ≤ 0.05) Tb.T compared to all other groups except HSA/PTHrP, dependent on Stro-1+ cell incorporation.

### Host cell/tissue invasion and mineralisation assessed by histological analysis

**Fig 2:**
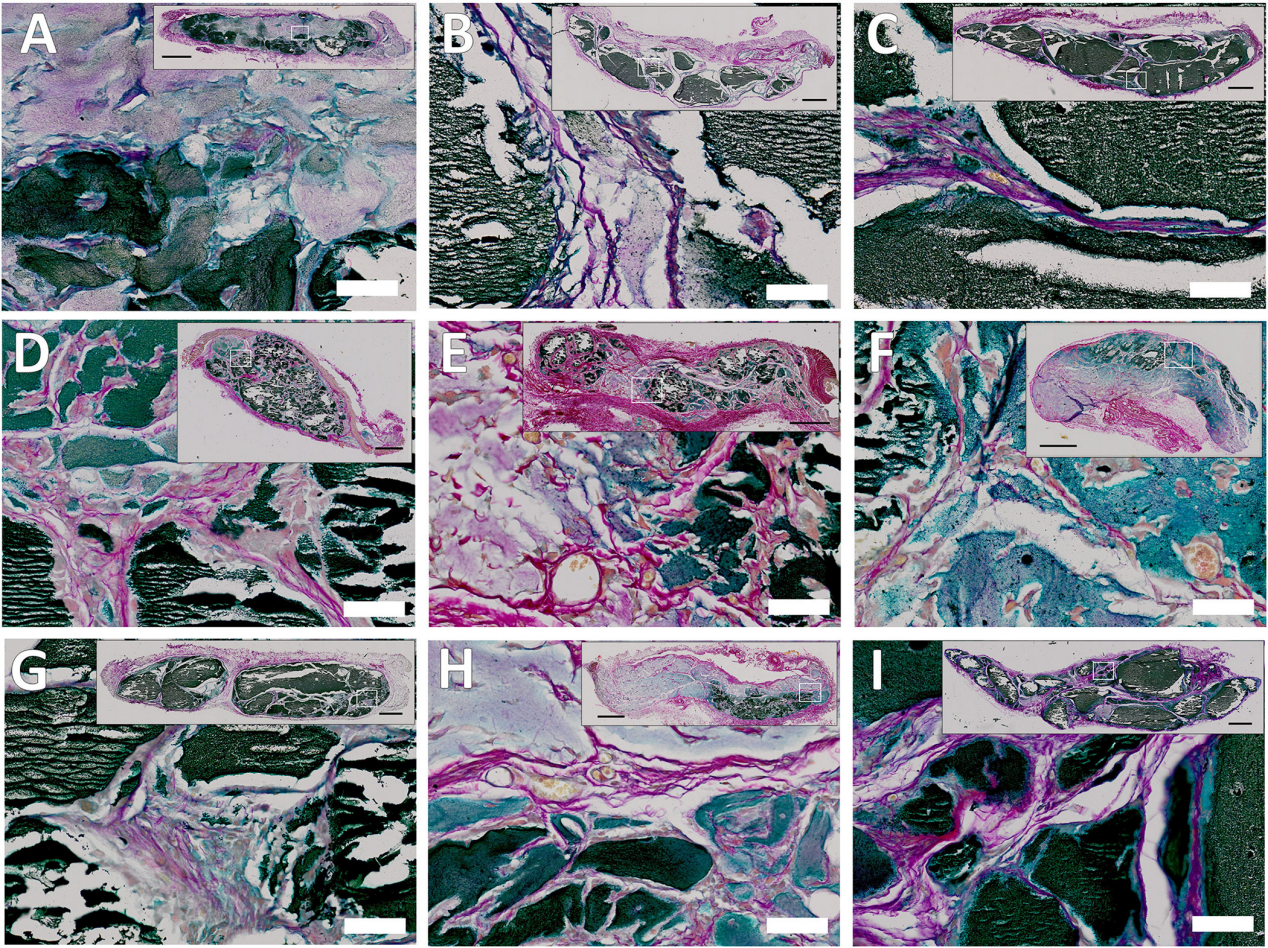
Hydrogel mineralisation between growth factor groups following 28 days in vivo implantation. Groups included irradiated ALG/ECM (**A**), ALG/Col (**B**), ALG/ECM (**C**), ALG/ECM HSA (**D**), ALG/ECM HSA/VEGF (**E**), ALG/ECM HSA/TGF-β3 (**F**), ALG/ECM HSA/BMP-2 (**G**), ALG/ECM HSA/PTHrP (**H**) and ALG/ECM HSA/VitD3 (**I**). Images were taken at high (scale bar is 500 μm) and low magnification (box inserts–scale bar is 50 μm).

Following micro CT analysis, hydrogels were assessed histologically to determine the degree of bone formation and host tissue invasion. A number of histological stains were assessed to highlight different components within the hydrogels. Following staining with Von Kossa, areas of mineralised tissue were observed in all hydrogel samples (Fig 2). Areas of dense black were uniformly torn and shredded across all samples, typical of mineralised bone upon sectioning. Clear differences could be seen between growth factor groups including reduced mineralisation within irradiated ALG/ECM, HSA/VEGF, HSA/TGF-β3 and HSA/PTHrP (Fig 2A, 2E, 2F and 2H, respective). Histological stains were subsequently quantified for comparison using a specialised Image J macro (S2A Fig). Control non-implanted hydrogels were also histologically assessed (S8 Fig). Interestingly, hydrogels stained strongly with Alcian blue (S8A Fig). No stain was observed within Von Kossa and Goldner’s Trichrome stained control hydrogels (S8B and S8C Fig). Incorporated Stro-1+ cells were observed homogeneously spread throughout each hydrogel (depicted by white arrows).

#### Alcian blue/Sirius red

**Fig 3:**
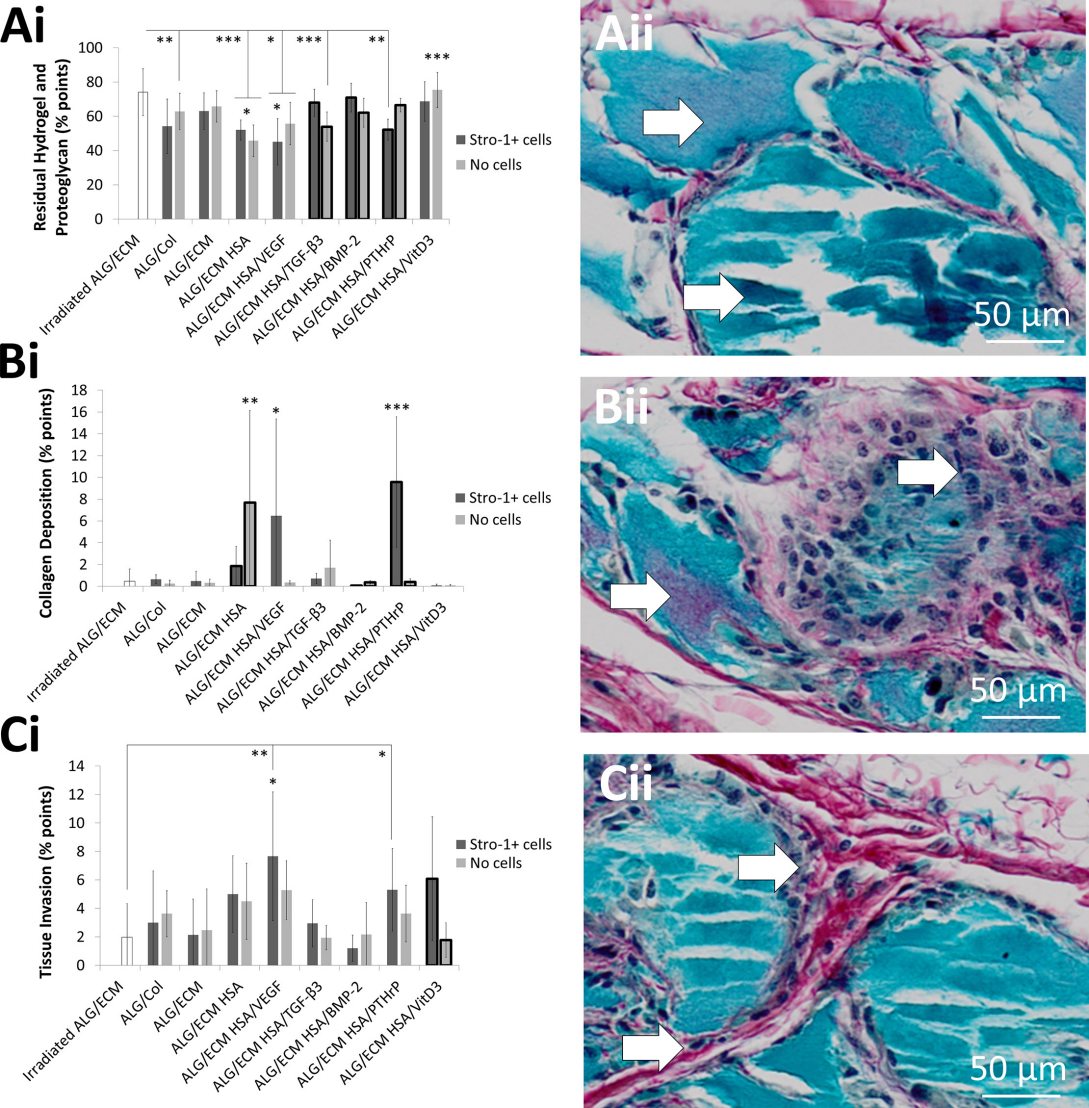
Histological analysis of hydrogels stained with Alcian blue/Sirius red. Hydrogels were subcutaneously implanted within immunodeficient for 28 days. Colour quantification was through the use of an optimised Image J macro (S2 Fig). Blue indicated proteoglycan deposition and residual hydrogel (**A**), purple indicated collagen deposition within the hydrogel (**B**) and red indicated tissue invasion (**C**). Emboldened columns depict statistically significant intragroup differences between those with and without Stro-1+ cell incorporation. Asterisks depict statistical difference between the group above which the asterisk is positioned and all the other groups; if positioned centrally above both groups with and without Stro-1+ cell incorporation, statistical difference was observed for both compared across all groups. Error bars are S.D. * P ≤ 0.05, ** P ≤ 0.01, *** P ≤ 0.001.

Within an Alcian blue and Sirius red section, blue areas typically denote proteoglycan deposition, however control non-implanted hydrogels were also heavily stained blue (S8A Fig). Consequently, blue quantification assessed both proteoglycan deposition and residual hydrogel following implantation (Fig 3A). Significantly (P ≤ 0.01) reduced proteoglycan deposition and residual hydrogel was observed within most growth factor groups excluding ALG/ECM, HSA/BMP-2 and HSA/VitD3 compared to irradiated ALG/ECM (S9A Fig). Control HSA demonstrated significantly (P ≤ 0.05) reduced proteoglycan deposition and residual hydrogel compared to all groups except HSA/VEGF, dependent on Stro-1+ cell incorporation. Indeed, most differences observed were dependent on Stro-1+ cell incorporation. Significant intra-group differences were observed within HSA/TGF-β3, HSA/BMP-2 and HSA/PTHrP groups.

Purple stain indicated areas of collagen deposition within the original hydrogel; usually collagen is red, but due to the intense blue stain of the original hydrogel, these areas appeared purple (Fig 3B). Only HSA *without*, and HSA/VEGF and HSA/PTHrP *with* Stro-1+ cells, exhibited significantly (P ≤ 0.05) greater collagen deposition compared to all other groups (S9B Fig). HSA/VitD3 exhibited significantly (P ≤ 0.01) lower collagen deposition compared to HSA dependent on Stro-1+ cell incorporation. Significant intra-group differences were observed within HSA, HSA/BMP-2 and HSA/PTHrP groups.

Red stained tissue, typically considered collagenous matrix, corresponded to host tissue invasion and was observed histologically as a fibrous network of threads infiltrating the hydrogel boundaries and occasionally spanning the width of the hydrogel (S2B and S2C Fig). Where blue colouration accounted for up to 80% of the hydrogel area, red accounted for ≤ 10%, similar to that for collagen deposition (Fig 3C). Overall, the three colours accounted for close to 100% of the hydrogel area. Minimal significant differences across groups were observed in comparison to control ALG/ECM. However, HSA/VEGF with Stro-1+ cell incorporation exhibited significantly (P ≤ 0.05) greater tissue invasion compared to irradiated ALG/ECM, ALG/ECM, HSA/TGF-β3 and HSA/BMP-2 (S8C Fig). Other significant inter-group differences included HSA/VitD3 compared to irradiated ALG/ECM, ALG/ECM and HSA/BMP-2, where HSA/VitD3 exhibited significantly (P ≤ 0.05) greater tissue invasion, dependent on Stro-1+ cell incorporation. Significant intra-group differences were only observed within the HSA/VitD3 group.

#### Von Kossa

**Fig 4:**
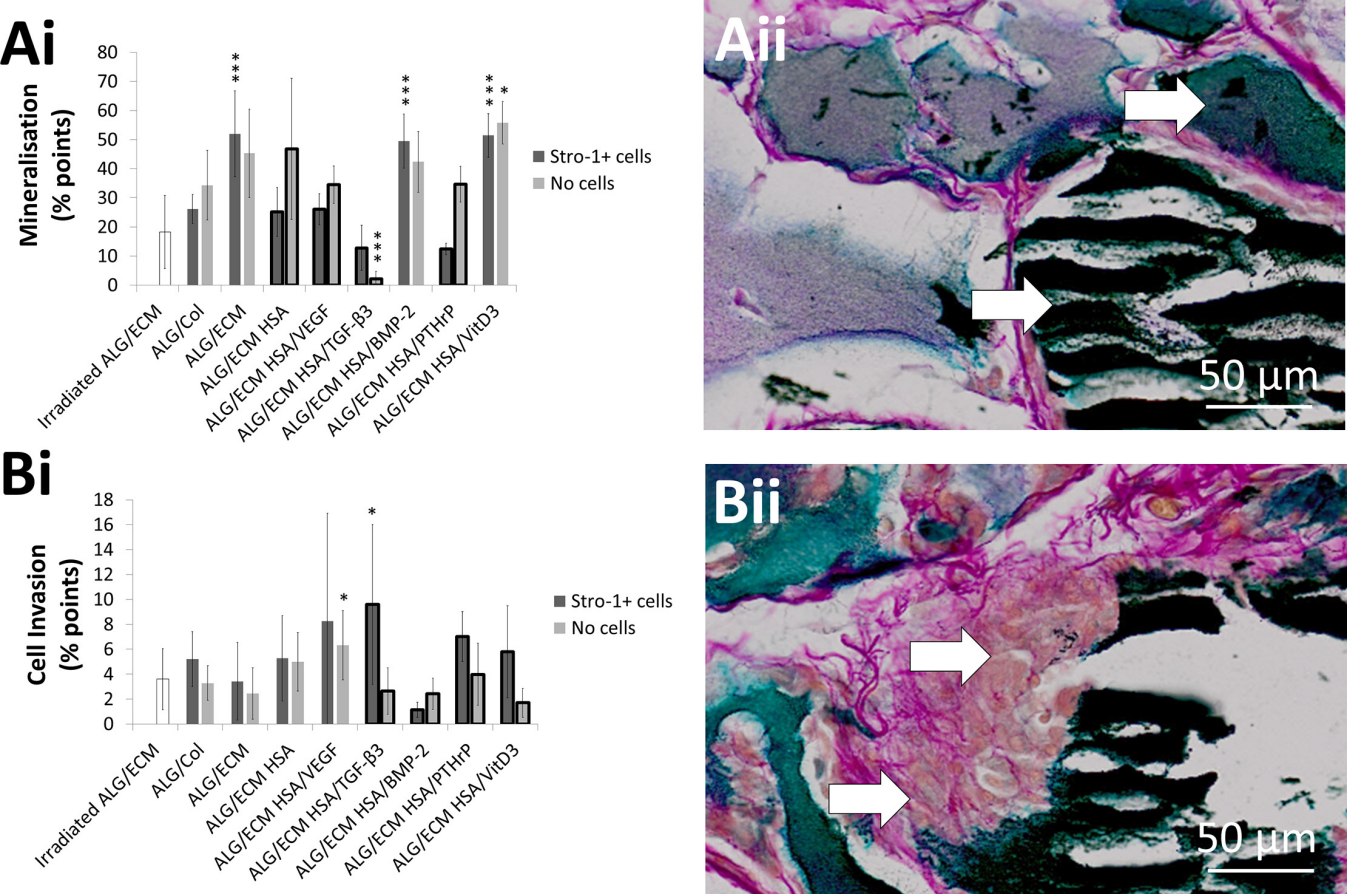
Histological analysis of hydrogels stained with Von Kossa. Hydrogels were subcutaneously implanted within immunodeficient mice for 28 days. Colour quantification was through the use of an optimised Image J macro (S2 Fig). Black indicates mineralised tissue (**A**) and pink indicates cell invasion (**B**). Emboldened columns depict statistically significant intragroup differences between those with and without Stro-1+ cell incorporation. Asterisks depict statistical difference between the group above which the asterisk is positioned and all the other groups; if positioned centrally above both groups with and without Stro-1+ cell incorporation, statistical difference was observed for both compared across all groups. Error bars are S.D. * P ≤ 0.05, ** P ≤ 0.01, *** P ≤ 0.001.

Mineralised tissue, indicative of new bone tissue formation, was confirmed by intense black stain following Von Kossa analysis within the hydrogels. ALG/ECM was not significantly different from either HSA/BMP-2 or HSA/VitD3, and all 3 groups exhibited significantly (P ≤ 0.001) increased mineralisation compared to all other groups including irradiated ALG/ECM (Fig 4A). Interestingly, results were very different between those groups with Stro-1+ cell incorporation and those without (S10A Fig). In the absence of Stro-1+ cells, only ALG/ECM and HSA/VitD3 exhibited enhanced mineralisation compared to most, but not all, other groups. Conversely, without Stro-1+ cell incorporation HSA/BMP-2 exhibited enhanced mineralisation in comparison to irradiated ALG/ECM. When Stro-1+ cells were not incorporated, HSA/TGF-β3 exhibited significantly (P ≤ 0.001) reduced mineralisation in comparison to all other groups. No significant intra-group difference were observed within ALG/ECM, HSA/BMP-2 and HSA/VitD3 groups, however significant (P ≤ 0.05) intra-group differences were observed in all other groups except ALG/Col.

Many dense areas which were damaged and shredded lay within the Von Kossa regions (black areas), typical of mineralised tissue during histological sectioning (S2Ci Fig). Consequently, these areas were included in quantification of black areas and assessed separately (S3Bi Fig). No growth factor groups exhibited significantly increased mineralisation compared to control ALG/ECM, with or without consideration of torn and shredded areas. Indeed, careful comparison revealed comparable trends between groups, independent of torn/shredded area quantification (Fig 4A, S3Bi, S3Bii and S9A Figs).

Pink colouration highlights cell cytoplasm and therefore provides an assessment of host cell invasion. In this instance the stain also highlights collagen (S2Ciii Fig) and therefore closely reflects red staining in Alcian blue/Sirius red. The results show similar, but not identical trends between both datasets (Figs 3C and 4B). HSA/TGF-β3 with Stro-1+ cell incorporation exhibited significantly (P ≤ 0.05) increased cell invasion compared to irradiated ALG/ECM, ALG/ECM and HSA/BMP-2. HSA/VEGF without Stro-1+ cell incorporation exhibited significantly (P ≤ 0.05) increased cell invasion compared to ALG/ECM, HSA/TGF-β3, HSA/BMP-2 and HSA/VitD3. Other inter-group differences included HSA/BMP-2 compared to HSA/VEGF both with Stro-1+ cells, and HSA compared to HSA/VitD3 both without Stro-1+ cells (S10C Fig). HSA/TGF-β3, HSA/BMP-2, HSA/PTHrP and HSA/VitD3 all exhibited significant (P ≤ 0.05) intra-group differences where Stro-1+ cell incorporation had a positive effect on cell invasion, except in HSA/BMP-2 groups.

#### Goldner’s Trichrome

**Fig 5:**
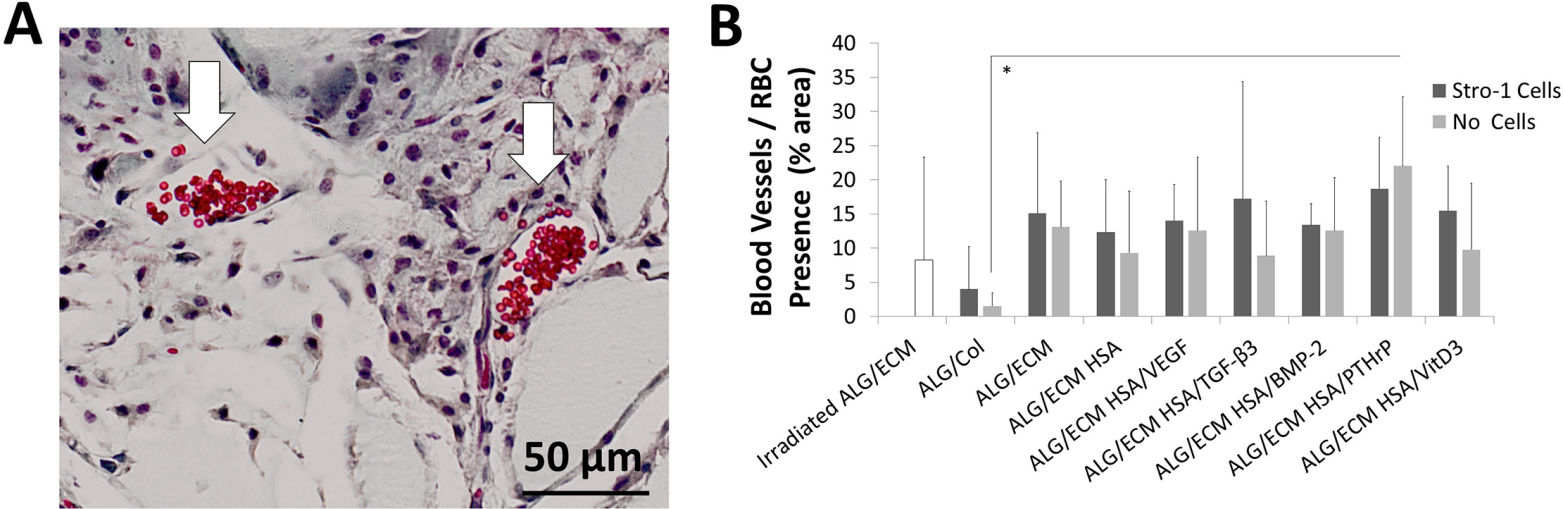
Histological analysis of hydrogels stained with Goldner’s Trichrome. Hydrogels were subcutaneously implanted within immunodeficient mice for 28 days. A square grid (200 μm) overlay was used to quantify erythrocytes (**A**) and assess vascularisation (**B**). Error bars are S.D. * P ≤ 0.05.

Erythrocytes stained bright red following histological processing for Goldner’s Trichrome (Fig 5A). Erythrocyte presence was used to assess hydrogel vascularisation by host tissue (Fig 5B and S11 Fig). No significant differences were observed between any two groups except irradiated ALG/ECM and HSA/PTHrP, without Stro-1+ cell incorporation (S12 Fig). No intra-group differences were observed. Positive immuno-labelling for vWF within and outside the hydrogels supported the identification of blood vessel formation (S13A and S13B Fig).

Following colour quantification, data across growth factor groups was collated and correlation analysis between stains was assessed (S14 Fig). Correlation analysis provided assessment of internal mechanisms during host invasion and cellular colonisation. Residual hydrogel and proteoglycan deposition decreased with host invasion. Importantly however, residual hydrogel did not correlate with mineralised tissue. Tissue invasion positively correlated with cell invasion, collagen deposition and vascularisation. Interestingly, tissue invasion did not correlate with mineralisation. Correlations were independent of Stro-1+ cell incorporation. However, only groups *with* Stro-1+ cell incorporation demonstrated positive correlation between collagen deposition and cell invasion/vascularisation, and negative correlation between collagen deposition/cell invasion and mineralisation.

Following *in vivo* culture, in an attempt to identify which tissues were contributed to by the implanted cells, sectioned samples were immuno-labelled with anti-human vimentin antibodies, a universally expressed marker on all possible differentiated cell types. However, the authors were unable to identify the implanted human Stro-1+ cells, possibly due to the low seeding density (S13C Fig). Work run in parallel with this study identified a lack of cell proliferation following implantation within these hydrogels [48, 49]; it is therefore conceivable that the implanted human cells perished and after *in vivo* culture there were minimal to no human cells remaining.

## Discussion

The current study has explored a multifactorial approach towards bone development and tissue engineering utilising ALG/ECM hydrogels *in vivo*. Growth factor loaded-microparticles and Stro-1+ SSCs were incorporated to increase mineralisation and further enhance bone formation. Interestingly, *all* hydrogel groups formed mineralised tissue following ectopic subcutaneous *in vivo* implantation for 28 days. Indeed no individual growth factor significantly enhanced mineralisation compared to control ALG/ECM, as a possible consequence of retained inherent growth factors within the bone ECM component [52, 64, 65]. This hypothesis is partially supported by reduced bone formation observed within irradiated ALG/ECM samples. Exposure to UV can cause growth factor degradation, but also alginate degradation and increased cross-linking. High levels of cross-linking within the ECM component as a result of UV irradiation has been shown to increase enzymatic resistance, a favourable property for load-bearing applications, such as bone tissue engineering [66]. γ-irradiation of alginate has been shown to significantly reduce polymerization undermining hydrogel stability [67]. However, a recent study has shown that UV irradiation did not affect mechanical properties within alginate scaffolds [68]. Consequently, reduced bone formation within UV irradiated hydrogels was due to inherent growth factor degradation. UV irradiation effects on the molecular structure of collagen within ECM component may also have contributed to these observations. Although not statistically significant, the numerical reduction in bone formation and vascularisation between ALG/ECM and ALG/Col also supports the hypothesis that ECM has inherent growth factors. Larger sample numbers and increased replicates may have helped these numerical differences reach statistical significance. The current results suggest bone ECM is an inexpensive, osteoinductive biomaterial with potential applications for tissue engineering and regenerative medicine, as observed in our previous *ex vivo* organotypic studies [48, 49].

Interestingly, enhanced bone formation was not observed within HSA/BMP-2 groups with Stro-1+ cell incorporation, compared to irradiated ALG/ECM. Without Stro-1+ cell incorporation HSA/BMP-2 exhibited significantly greater bone formation, similar to VitD3, compared to irradiated ALG/ECM; potentially through formation of thicker trabeculae. Indeed, these significant differences in comparison to irradiated ALG/ECM highlighted the inherent osteoinductive efficacy of bone ECM, as possible growth factor degradation via irradiation significantly decreased bone formation. Importantly, the osteoinductive capacity of control ALG/ECM was such that neither osteogenic factor, BMP-2 nor VitD3, could further enhance bone formation. The bone ECM component was observed to be highly osteoinductive possibly due to inherent growth factors. Addition of osteogenic factors at the concentrations employed in these studies did not further enhance bone formation.

Increased bone formation was in part, dependent on incorporation of Stro-1+ cells. Therapeutic SSC incorporation can reconstitute microenvironmental cues within hydrogels through their secretome (growth factors, micro-vesicles, and exosomes released into the extracellular milieu) [69]. Combination of SSCs within several collagen-based hydrogels has previously yielded improved osteochondral tissue formation [70–75]. Although BMP-2 and VitD3 demonstrated similarly increased bone formation, their response to cell incorporation was quite different. Interaction of BMP-2 and Stro-1+ cells led to decreased bone formation, whilst interaction with VitD3 increased bone formation. PBV takes into consideration TV and therefore formation of potential pre-mineralised tissues such as osteoid. Enhanced bone formation within HSA/BMP-2, unlike HSA/VitD3, was dependent on the absence of Stro-1+ cell incorporation, and significant intra-group difference suggests the presence of Stro-1+ cells did not augment BMP-2 induced bone formation. It is possible that Stro-1+ cells were highly responsive to BMP-2 signalling and differentiated without proliferation; less cells, less bone formation. Alternatively, and possibly more accurately, the presence of Stro-1+ cells may have limited or inhibited BMP-2 induced bone formation.

Chondrogenic HSA/TGF-β3 groups did not enhance cartilage formation, possibly due to inadequate concentration, spatiotemporal delivery, or reduced cells evidenced by the lack of increased total TV and proteoglycan deposition. Our studies indicate TGF-β3 inhibited bone formation; in marked contrast to the osteoinductive effect of ALG/ECM. Further micro CT analysis revealed that TGF-β3 delivery affected bone structure increasing the number of bone trabeculae compared to HSA/BMP-2 and HSA/VitD3, which in turn significantly increased BS/BV values. It is generally accepted that higher numbers of thick trabeculae provide the greatest structural strength in 3D bone tissue and therefore reduced Tb.N and high BS/BV following TGF-β3 signalling resulted in lower bone formation of potentially inferior mechanical strength. These structural differences were only significant when hydrogels were seeded with Stro-1+ cells, suggesting the cells amplified the effect of TGF-β3, again through potential inhibitory and counter-osteogenic signalling. VEGF and PTHrP did not significantly affect bone formation or bone structure according to micro CT data, possibly due to resolution limitations with a scan at 18 μm resolution; if VEGF and PTHrP affected bone formation but on a relatively smaller scale than other growth factors, these changes may not have been resolved at 18 μm resolution. Alternatively, extensive host-driven vascularisation in all groups may have superseded any effect from implanted VEGF; supported by the lack of difference in vascularisation between groups. PTHrP may not have been present at sufficient concentrations to elicit an observable effect on bone formation.

All hydrogel groups investigated exhibited areas of mineralised tissue following Von Kossa staining indicating capacity for bone formation. Subsequent to these studies Lee C. S. *et al* [76] reported on passive calcification of alginate microbeads following *in vivo* implantation subcutaneously within mice. However, quantification of torn/shredded calcified alginate areas within our hydrogel implants did not significantly affect observed differences between groups, nor did residual hydrogel correlate with mineralisation/calcification. To avoid issues with passive calcification in future, alginate cross-linking with barium chloride rather than calcium chloride would be optimal. The histological trends reflected those observed from micro CT analysis, where HSA/BMP-2, HSA/VitD3 and control ALG/ECM exhibited the greatest mineralised tissue formation, but were not significantly different from each other. Osteogenic growth factors did not further enhance bone formation within our hydrogels due to the inherent osteoinductive capacity of the bone ECM component. In support, irradiated ALG/ECM exhibited significantly reduced mineralisation through possible degradation of the inherent growth factors, although other effects of UV irradiation on the molecular structure of collagen within the ECM component may have contributed to these reductions. Incorporation of inducible Stro-1+ cells was important for bone formation, dependent on the growth factor present. Delivery alongside angiogenic or chondrogenic growth factors actively reduced bone formation following histological assessment compared with micro CT analysis. Importantly, these same groups without cell incorporation did not demonstrate the same reduction in bone formation.

Extensive host tissue invasion within *all* hydrogel samples hindered analysis of tissue formation originating directly from incorporated Stro-1+ cells. Indeed, cell invasion positively correlated with host tissue invasion, appearing as a cell dense collagenous network throughout the hydrogel structure. Within angiogenic and chondrogenic groups, soft tissue formation either replaced original hydrogel during potential resorption, or contributed to the original hydrogel volume restructuring internal composition. However, since residual hydrogel negatively correlated with host invasion, the former suggestion appears most likely. In general, ALG/ECM and osteogenic groups exhibited increased mineralisation without change to hydrogel volume, again supporting replacement rather than addition. Bone ECM provided both hydrogel invasion points and internal structural conduits for host cell migration. Host blood vessels migrated into all hydrogel samples together with aggressive tissue and cell invasion, minimising inter-group differences. However, quantification of vascularisation was based on erythrocyte presence, which does not accurately reflect the number or size of blood vessels, only presence.

Although the hydrogel system described here has shown value for *in vivo* bone formation, interpretation to the clinical setting requires further study. The hydrogel system demonstrated capacity for bone formation *in vivo* even in the ectopic location utilised here. However, this also highlights a major limitation of the study, as subcutaneous implantation lacks the mechanical stresses that the hydrogel system would need to experience/perform under in the clinical arena. A second limitation is the fact the hydrogel system was implanted within immuno-compromised mice. Although alginate has been used in the clinical setting for a number of decades (e.g. wound dressings) further investigation would be required to ascertain whether the hydrogel system would elicit an immune response.

## Conclusion

The current studies demonstrate the application of an ALG/ECM hydrogel system for *in vivo* bone formation. The bone ECM component exhibited inherent osteoinductive capacity leading to enhanced mineralisation which was not further enhanced with additional growth factor concentrations applied in this study. Structural stabilisation of liquid ECM such that the hydrogel could be shaped and manipulated was afforded by combination with alginate. Bone formation within the hydrogel system could be altered with chondrogenic growth factors such that the system formed greater soft tissue. Taken together, ALG/ECM offers a bioactive alternative scaffold for utilisation within regenerative medicine, which may be further tailored, ultimately, to form the tissue of choice through incorporation of select growth factor-loaded microparticles. This study has shown the potential of a multifaceted approach to bone development and tissue engineering.

## Supporting Information

S1 Fig Micro CT analysis of intact adult mouse limb (forearm). Greyscale threshold at 80–255 (**A**) shows only mineralised tissue, whilst 60–255 (**B**) shows both mineralised and soft tissue.
(TIF)

S2 Fig Histological analysis of hydrogels implanted within immunodeficient mice for 28 days. A specialised macro on Image J was designed to quantify colours on histology images (**A**). First, the hydrogel was selected by drawing a ‘region of interest’ (**Ai**). ‘Colour threshold’ was then used to create a black and white mask (**Aii**). A point grid overlaid on the mask was used to quantify the colour (**Aiii**). Images were taken at low (**i**), medium (**ii**) and high magnification (**iii**). Consecutive sections were stained with Alcian blue/Sirius red (**B**), Von Kossa (**C**) and Goldner’s Trichrome (**D**). White arrows depict colour that have been quantified on each histological stain.
(TIF)

S3 Fig Histological analysis of torn/shredded areas within hydrogel samples following Alcian blue/Sirius red (A) and Von Kossa stain (B). Hydrogels were subcutaneously implanted within immunodeficient for 28 days. Colour quantification was through the use of an optimised Image J macro (S2 Fig). Blue indicated proteoglycan deposition and residual hydrogel (**Ai**) and black indicated mineralisation (**Bi**). Comparison between all groups with Stro-1+ cells (upper right corner–light grey) or without Stro-1+ cells (lower left corner–medium grey) were assessed by a One Way ANOVA with Tukeys post-hoc test (**ii**). Dark grey boxes depict t-test comparisons within groups between those with and without Stro-1+ cells. Emboldened columns depict statistically significant intragroup differences between those with and without Stro-1+ cell incorporation. Asterisks depict statistical difference between the group above which the asterisk is positioned and all the other groups; if positioned centrally above both groups with and without Stro-1+ cell incorporation, statistical difference was observed for both compared across all groups. Red box indicates non-comparison as irradiated ALG/ECM did not have Stro-1+ cells incorporated. NS indicates ‘no significance’. * P ≤ 0.05, ** P ≤ 0.01, *** P ≤ 0.001.
(TIF)

S4 Fig Hydrogels following harvest from immunodeficient mice after 28 days implantation. Scale bar is 5 mm.
(TIF)

S5 Fig Statistical analysis of micro CT data between growth factor groups with Stro-1+ cell incorporation. All data was analysed using One Way ANOVA with Tukeys post-hoc test. Tables separate into upper right and lower left corners detailing individual comparisons between all groups regarding the parameter stated adjacent. ‘NS’ indicates no significance. * P ≤ 0.05, ** P ≤ 0.01, *** P ≤ 0.001.
(TIF)

S6 Fig Statistical analysis of micro CT data between growth factor groups without Stro-1+ cell incorporation. All data was analysed using One Way ANOVA with Tukeys post-hoc test. Tables separate into upper right and lower left corners detailing individual comparisons between all groups regarding the parameter stated adjacent. ‘NS’ indicates no significance. * P ≤ 0.05, ** P ≤ 0.01, *** P ≤ 0.001.
(TIF)

S7 Fig Statistical analysis of micro CT data between those groups with and without Stro-1+ cell incorporation. NS indicates ‘no significance’. * P ≤ 0.05, ** P ≤ 0.01.
(TIF)

S8 Fig Histological analysis of control non-implanted hydrogels with Stro-1+ cell incorporation stained with Alcian blue/Sirius red (A), Von Kossa (B), and Goldner’s Trichrome (C). Images were taken at low (**i**, scale bar is 500 μm) and high (**ii**, scale bar is 100 μm) magnification.
(TIF)

S9 Fig Statistical analysis of histology data between growth factor groups from Alcian blue/Sirius red stained sections. Residual hydrogel and proteoglycan deposition (**A**), collagen deposition (**B**) and tissue invasion (**C**) were each statistically analysed. Comparison between all groups with Stro-1+ cells (upper right corner–light grey) or without Stro-1+ cells (lower left corner–medium grey) were assessed by a One Way ANOVA with Tukeys post-hoc test. Dark grey boxes depict t-test comparisons within groups between those with and without Stro-1+ cells. Red box indicates non-comparison as irradiated ALG/ECM did not have Stro-1+ cells incorporated. NS indicates ‘no significance’. * P ≤ 0.05, ** P ≤ 0.01, *** P ≤ 0.001.
(TIF)

S10 Fig Statistical analysis of histology data between growth factor groups from Von Kossa stained sections. Mineralisation (**A**) and cell invasion (**B**) were both statistically analysed. Comparison between all groups with Stro-1+ cells (upper right corner–light grey) or without Stro-1+ cells (lower left corner–medium grey) were assessed by a One Way ANOVA with Tukeys post-hoc test. Dark grey boxes depict t-test comparisons within groups between those with and without Stro-1+ cells. Red box indicates non-comparison as irradiated ALG/ECM did not have Stro-1+ cells incorporated. NS indicates ‘no significance’. * P ≤ 0.05, ** P ≤ 0.01, *** P ≤ 0.001.
(TIF)

S11 Fig Extensive vascularisation throughout the implanted hydrogel structure depicted by the presence of erythrocytes. Host blood vessel invasion is depicted by white arrows within magnified areas. Image was taken from a GT stained ALG/ECM hydrogel following 28 days *in vivo* implantation.
(TIF)

S12 Fig Statistical analysis of hydrogels stained with Goldner’s Trichrome. Comparisons between all groups with Stro-1+ cells (upper right corner–light grey) or without Stro-1+ cells (lower left corner–medium grey) were assessed by a One Way ANOVA with Tukeys post-hoc test. Dark grey boxes depict comparisons within groups between those with and without Stro-1+ cells. Red box indicates non-comparison as irradiated ALG/ECM did not have Stro-1+ cells incorporated. NS indicates ‘no significance’. * P ≤ 0.05.
(TIF)

S13 Fig Blood vessel formation and identification of human tissue. Positive red-brown staining for vWF (depicted by white arrows) identified blood vessel formation in the mouse tissue surrounding the hydrogel (**A**) and in the tissues invading the hydrogel (**B**). Samples stained for human-specific vimentin did not identify implanted Stro-1 cells (**C**). Sections were counterstained with haematoxylin. Scale bars measure 100 μm.
(JPG)

S14 Fig Statistical analysis of correlations between histologically assessed parameters. Following colour quantification of A/S, VK and GT stained samples, data for residual hydrogel and proteoglycan deposition (blue), tissue invasion (red), collagen deposition (purple), mineralisation (black), cell invasion (pink) and vascularisation (RBC presence) was combined across all growth factor groups and correlations between colours were assessed. Top right graphs depict correlations between all groups with Stro-1+ cell incorporation. Bottom left graphs depict correlations between all groups without Stro-1+ cell incorporation. Trend lines highlight those graphs depicting significant correlation. NS indicates ‘no significance’. ‡ denotes positive correlation. * P ≤ 0.05, ** P ≤ 0.01, *** P ≤ 0.001.
(JPG)
