# Supplementary figures and images for: In Vivo Assessment of Bone Regeneration in Alginate/Bone ECM Hydrogels with Incorporated Skeletal Stem Cells and Single Growth Factors
Source: PLoS One. 2015 Dec 16;10(12):e0145080. doi: 10.1371/journal.pone.0145080 (PMC4684226; doi:10.1371/journal.pone.0145080)

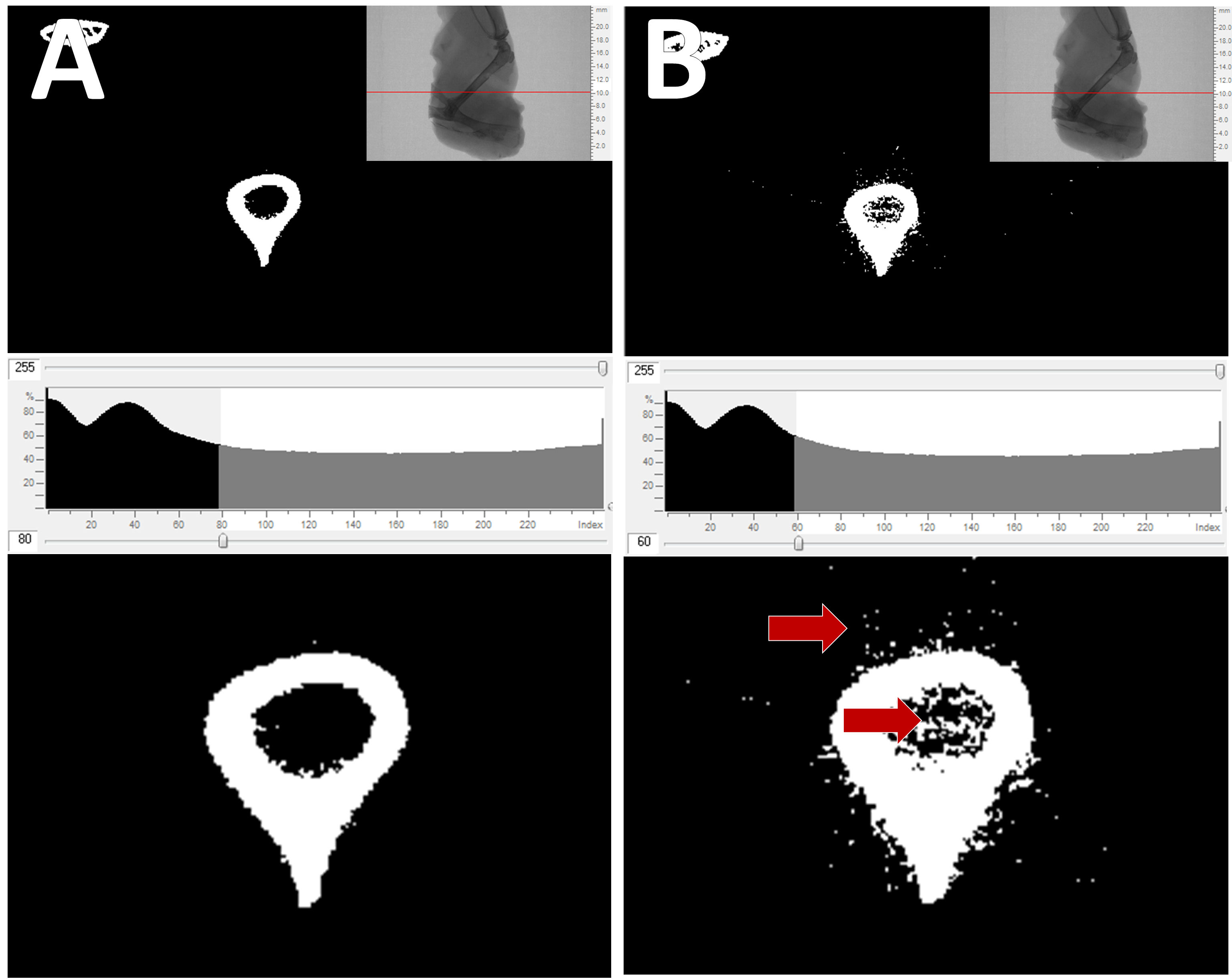

Supplement: S1 Fig — Greyscale threshold at 80–255 (A) shows only mineralised tissue, whilst 60–255 (B) shows both mineralised and soft tissue. (TIF) [file pone.0145080.s001.tif]

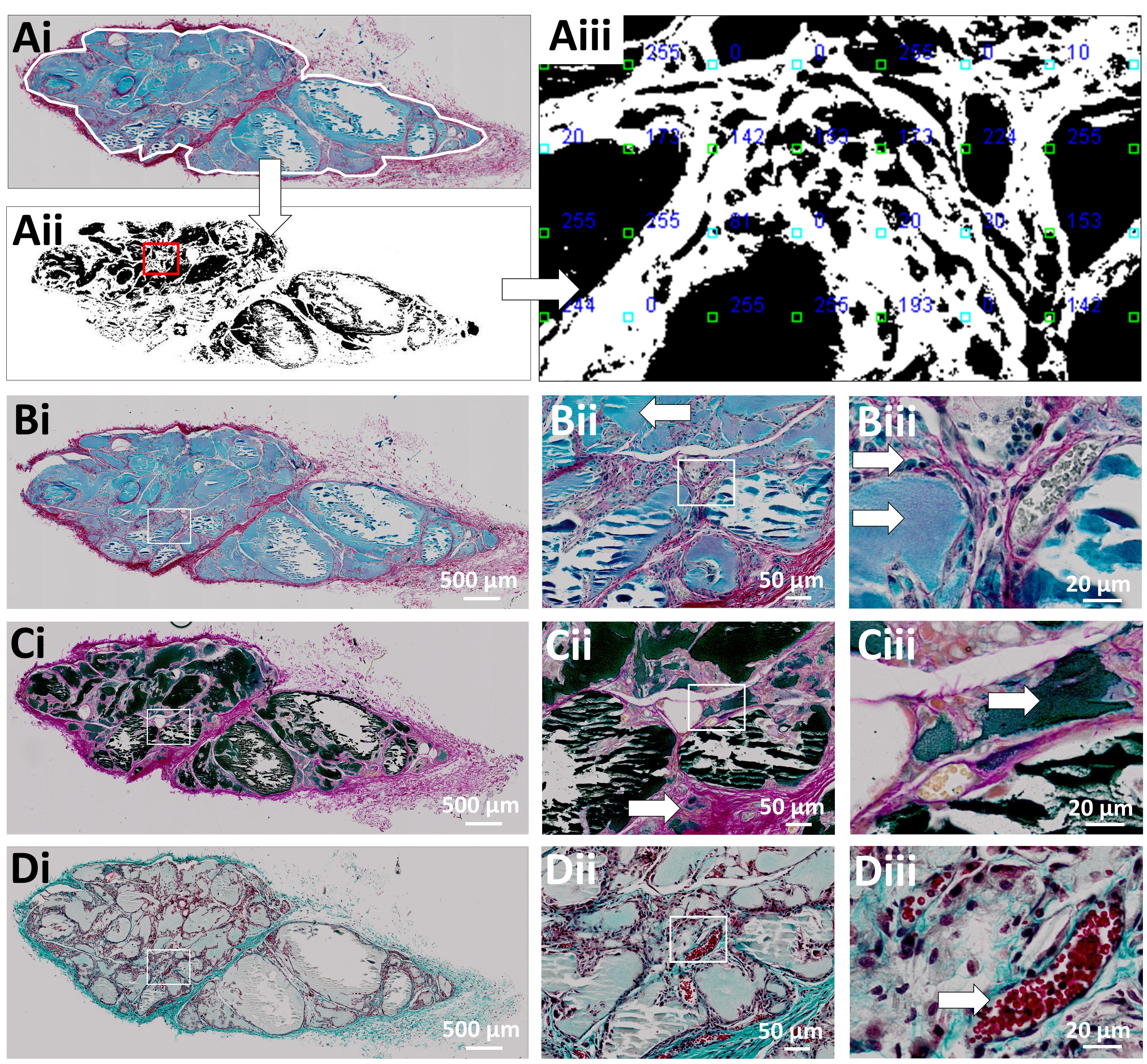

Supplement: S2 Fig — A specialised macro on Image J was designed to quantify colours on histology images (A). First, the hydrogel was selected by drawing a ‘region of interest’ (Ai). ‘Colour threshold’ was then used to create a black and white mask (Aii). A point grid overlaid on the mask was used to quantify the colour (Aiii). Images were taken at low (i), medium (ii) and high magnification (iii). Consecutive sections were stained with Alcian blue/Sirius red (B), Von Kossa (C) and Goldner’s Trichrome (D). White arrows depict colour that have been quantified on each histological stain. (TIF) [file pone.0145080.s002.tif]

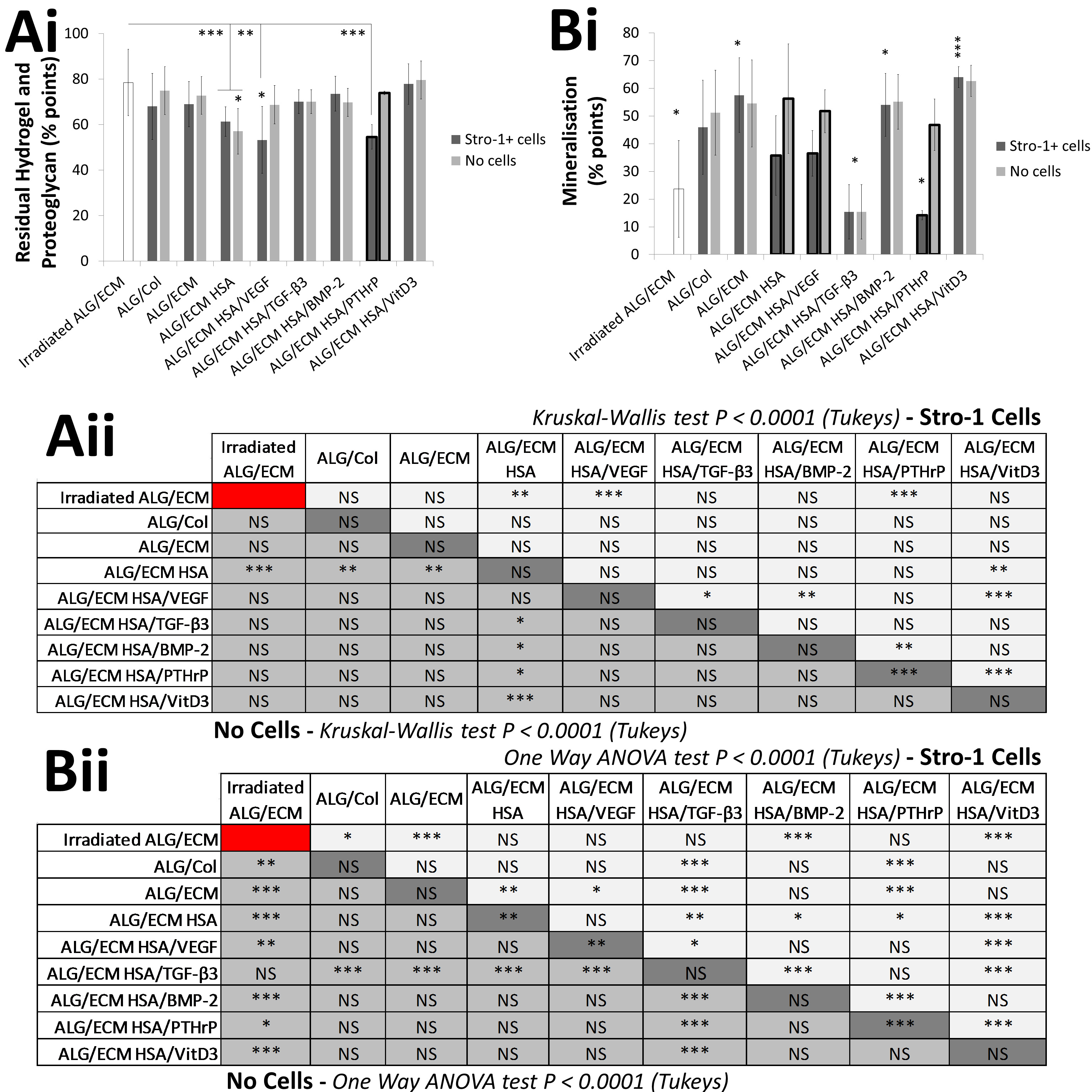

Supplement: S3 Fig — Hydrogels were subcutaneously implanted within immunodeficient for 28 days. Colour quantification was through the use of an optimised Image J macro (S2 Fig). Blue indicated proteoglycan deposition and residual hydrogel (Ai) and black indicated mineralisation (Bi). Comparison between all groups with Stro-1+ cells (upper right corner–light grey) or without Stro-1+ cells (lower left corner–medium grey) were assessed by a One Way ANOVA with Tukeys post-hoc test (ii). Dark grey boxes depict t-test comparisons within groups between those with and without Stro-1+ cells. Emboldened columns depict statistically significant intragroup differences between those with and without Stro-1+ cell incorporation. Asterisks depict statistical difference between the group above which the asterisk is positioned and all the other groups; if positioned centrally above both groups with and without Stro-1+ cell incorporation, statistical difference was observed for both compared across all groups. Red box indicates non-comparison as irradiated ALG/ECM did not have Stro-1+ cells incorporated. NS indicates ‘no significance’. * P ≤ 0.05, ** P ≤ 0.01, *** P ≤ 0.001. (TIF) [file pone.0145080.s003.tif]

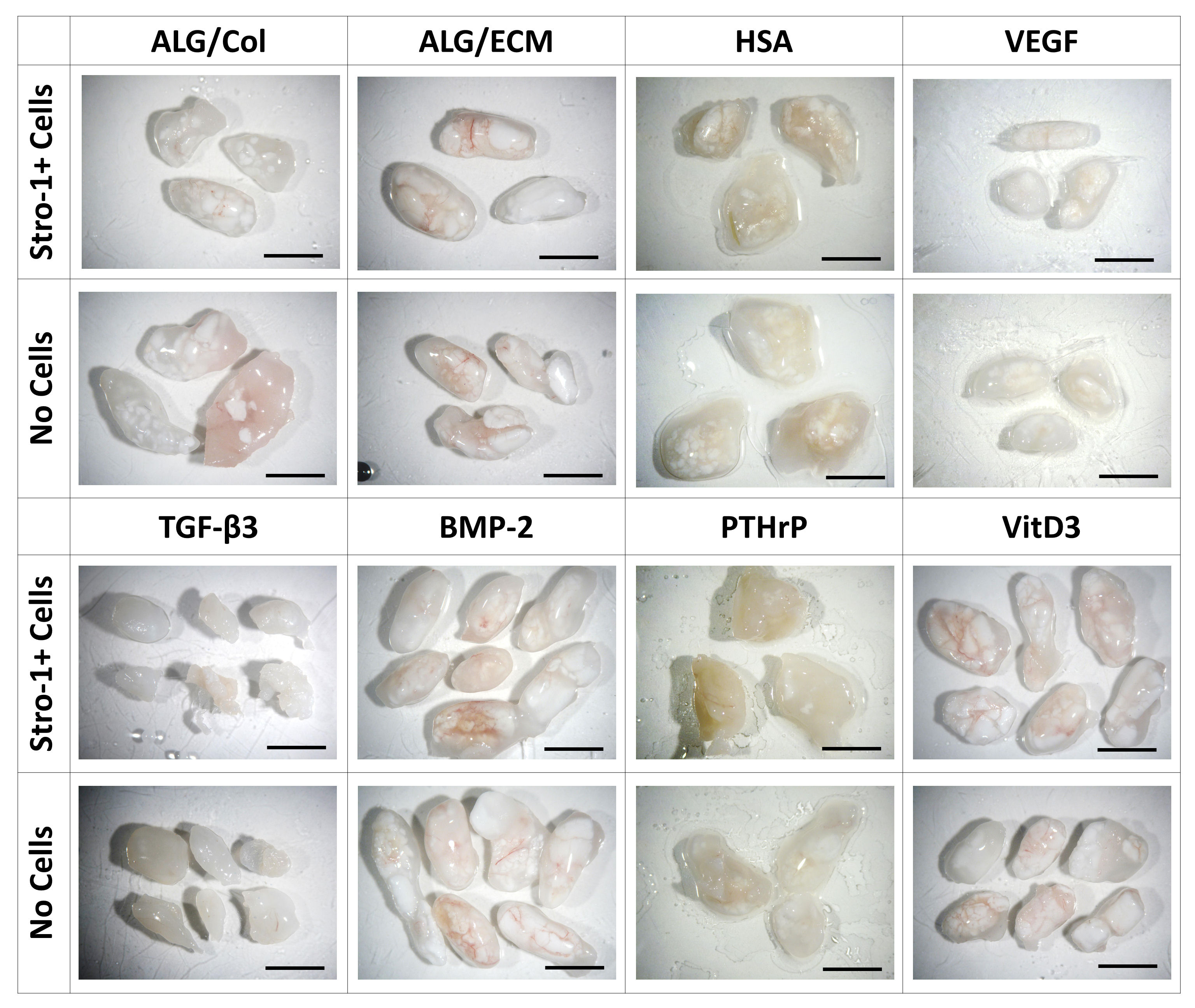

Supplement: S4 Fig — Scale bar is 5 mm. (TIF) [file pone.0145080.s004.tif]

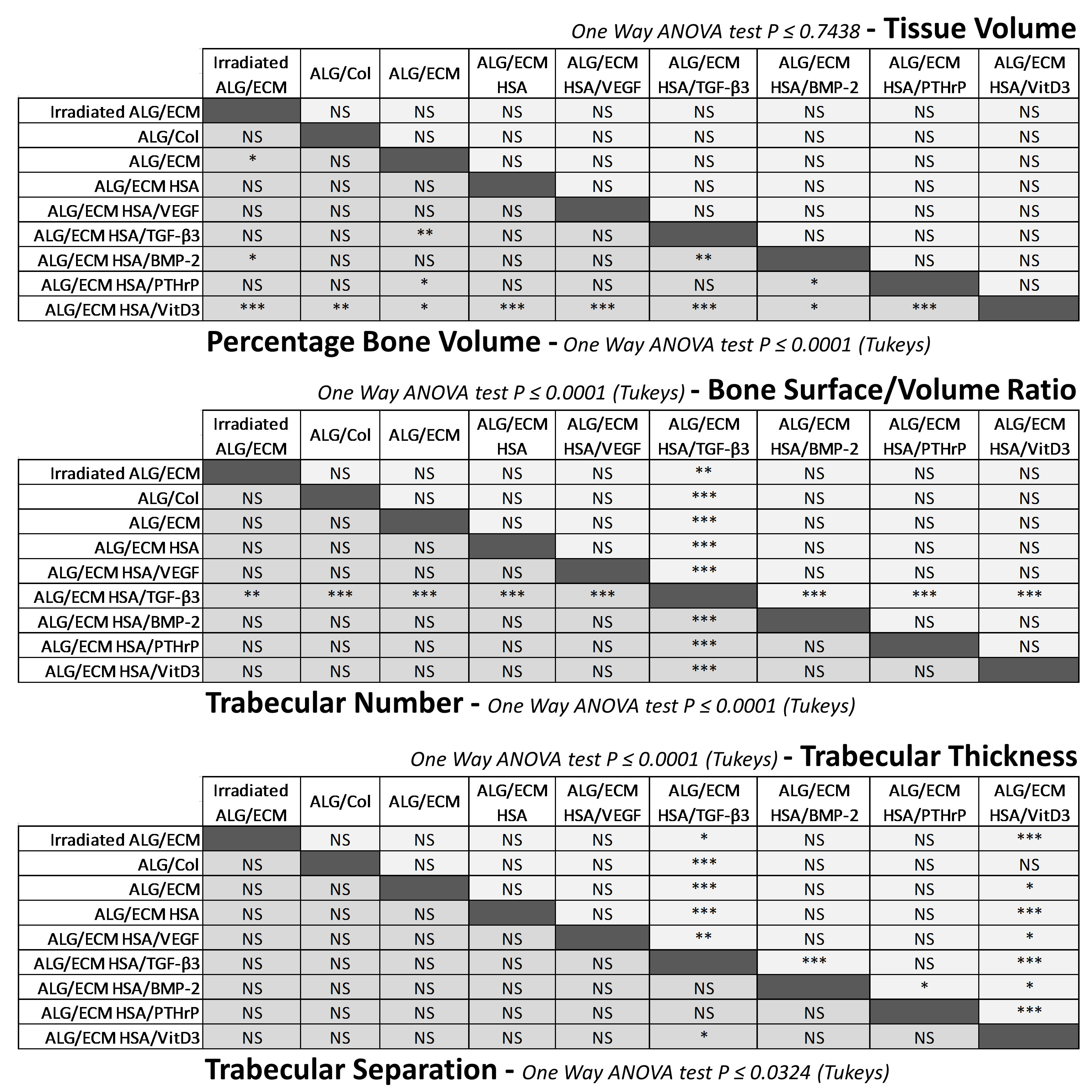

Supplement: S5 Fig — All data was analysed using One Way ANOVA with Tukeys post-hoc test. Tables separate into upper right and lower left corners detailing individual comparisons between all groups regarding the parameter stated adjacent. ‘NS’ indicates no significance. * P ≤ 0.05, ** P ≤ 0.01, *** P ≤ 0.001. (TIF) [file pone.0145080.s005.tif]

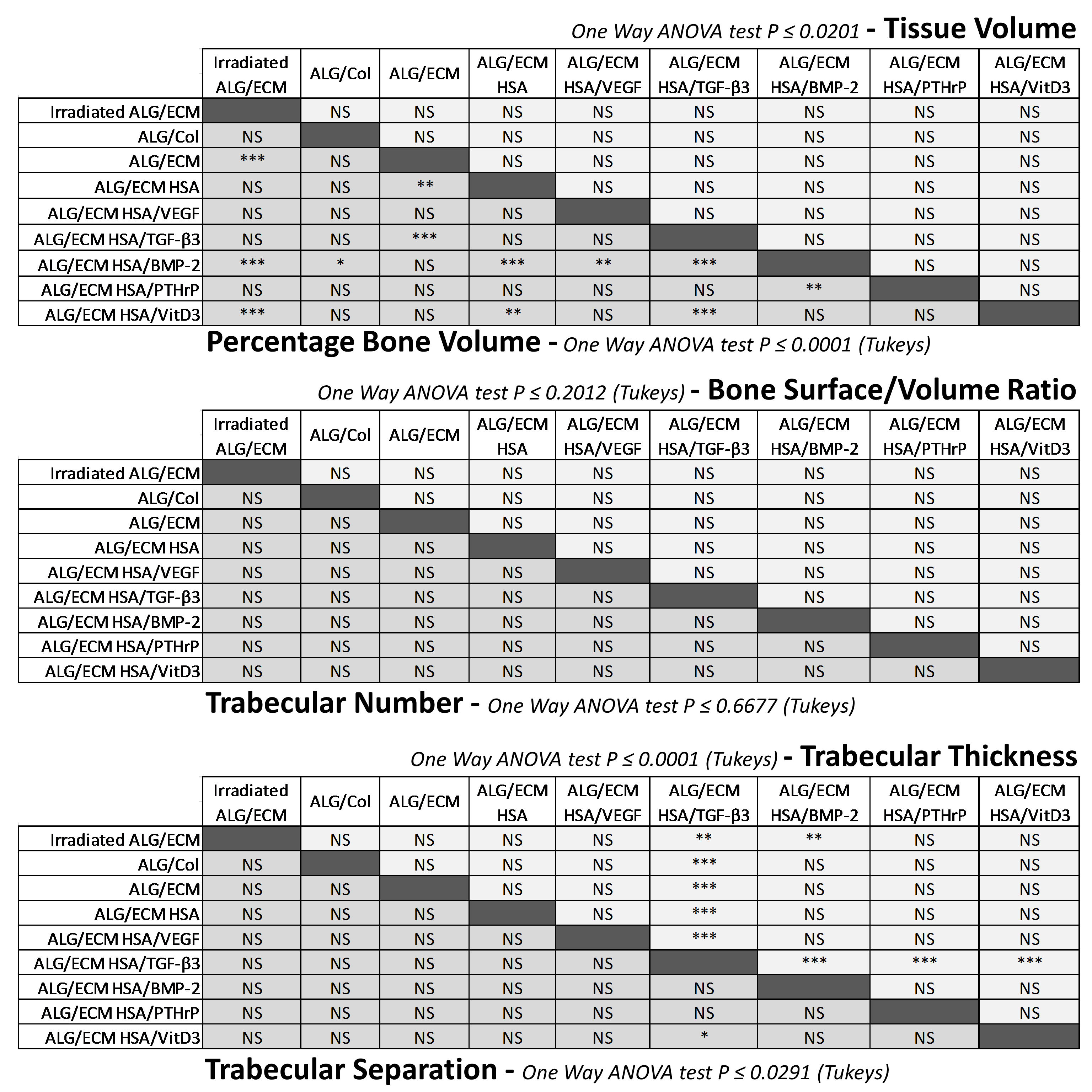

Supplement: S6 Fig — All data was analysed using One Way ANOVA with Tukeys post-hoc test. Tables separate into upper right and lower left corners detailing individual comparisons between all groups regarding the parameter stated adjacent. ‘NS’ indicates no significance. * P ≤ 0.05, ** P ≤ 0.01, *** P ≤ 0.001. (TIF) [file pone.0145080.s006.tif]

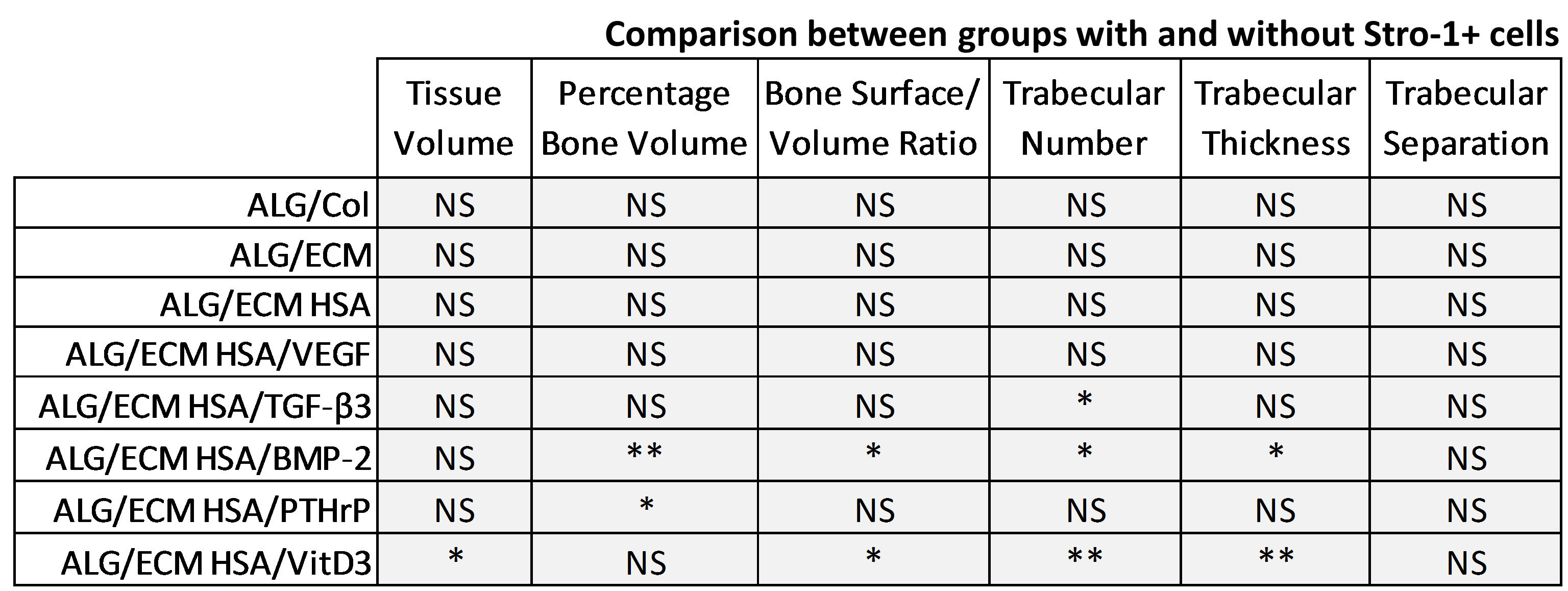

Supplement: S7 Fig — NS indicates ‘no significance’. * P ≤ 0.05, ** P ≤ 0.01. (TIF) [file pone.0145080.s007.tif]

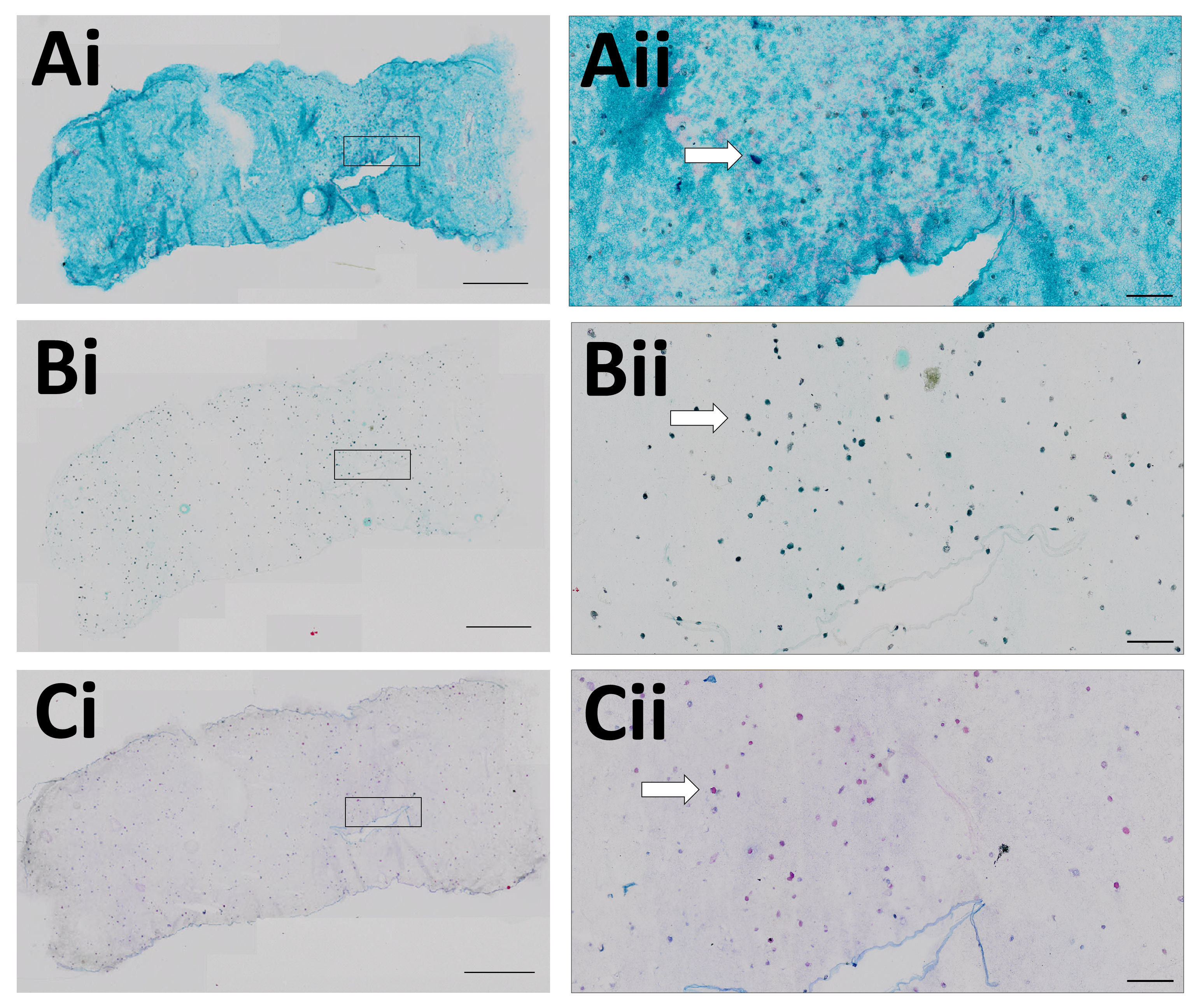

Supplement: S8 Fig — Images were taken at low (i, scale bar is 500 μm) and high (ii, scale bar is 100 μm) magnification. (TIF) [file pone.0145080.s008.tif]

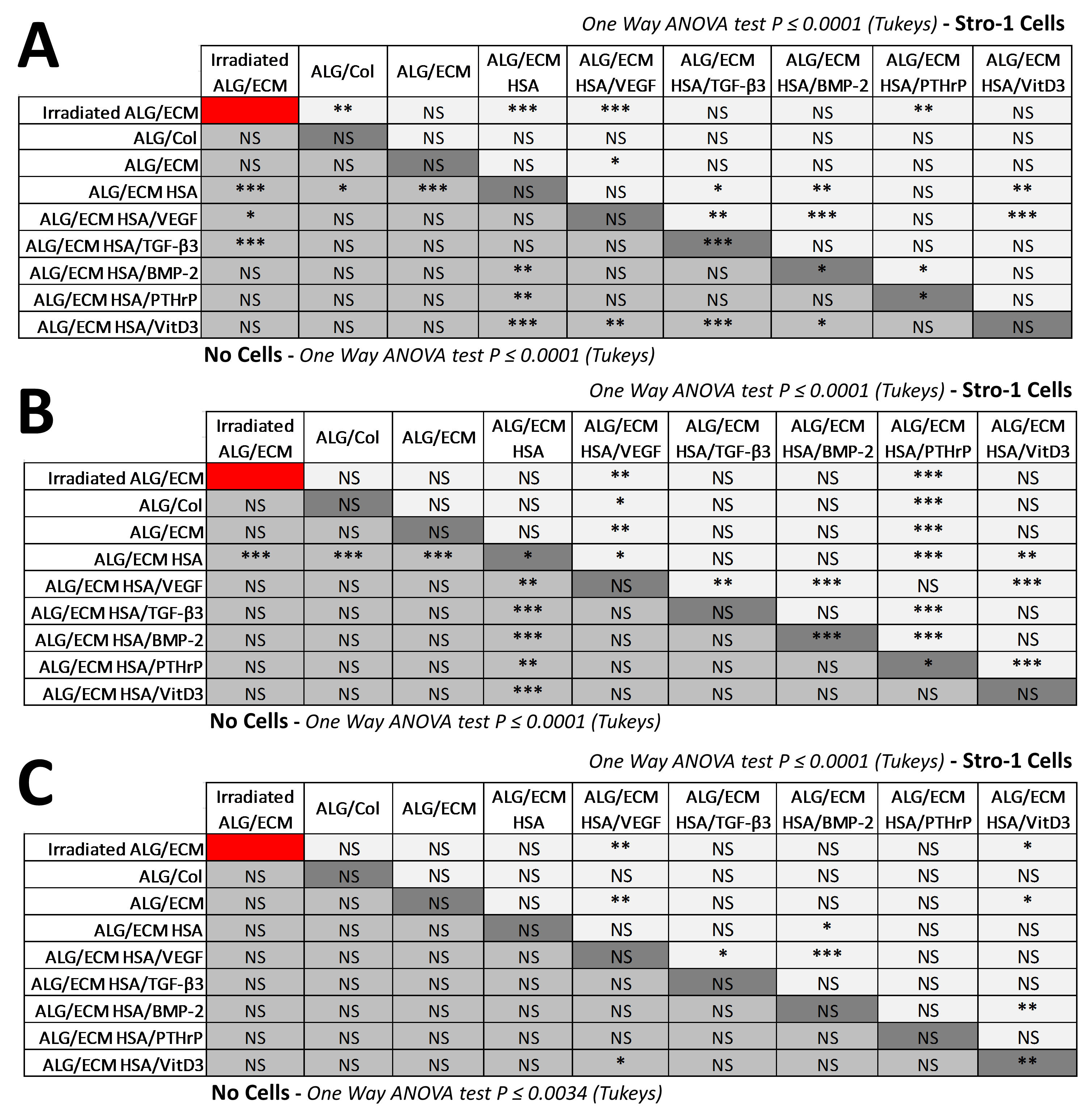

Supplement: S9 Fig — Residual hydrogel and proteoglycan deposition (A), collagen deposition (B) and tissue invasion (C) were each statistically analysed. Comparison between all groups with Stro-1+ cells (upper right corner–light grey) or without Stro-1+ cells (lower left corner–medium grey) were assessed by a One Way ANOVA with Tukeys post-hoc test. Dark grey boxes depict t-test comparisons within groups between those with and without Stro-1+ cells. Red box indicates non-comparison as irradiated ALG/ECM did not have Stro-1+ cells incorporated. NS indicates ‘no significance’. * P ≤ 0.05, ** P ≤ 0.01, *** P ≤ 0.001. (TIF) [file pone.0145080.s009.tif]

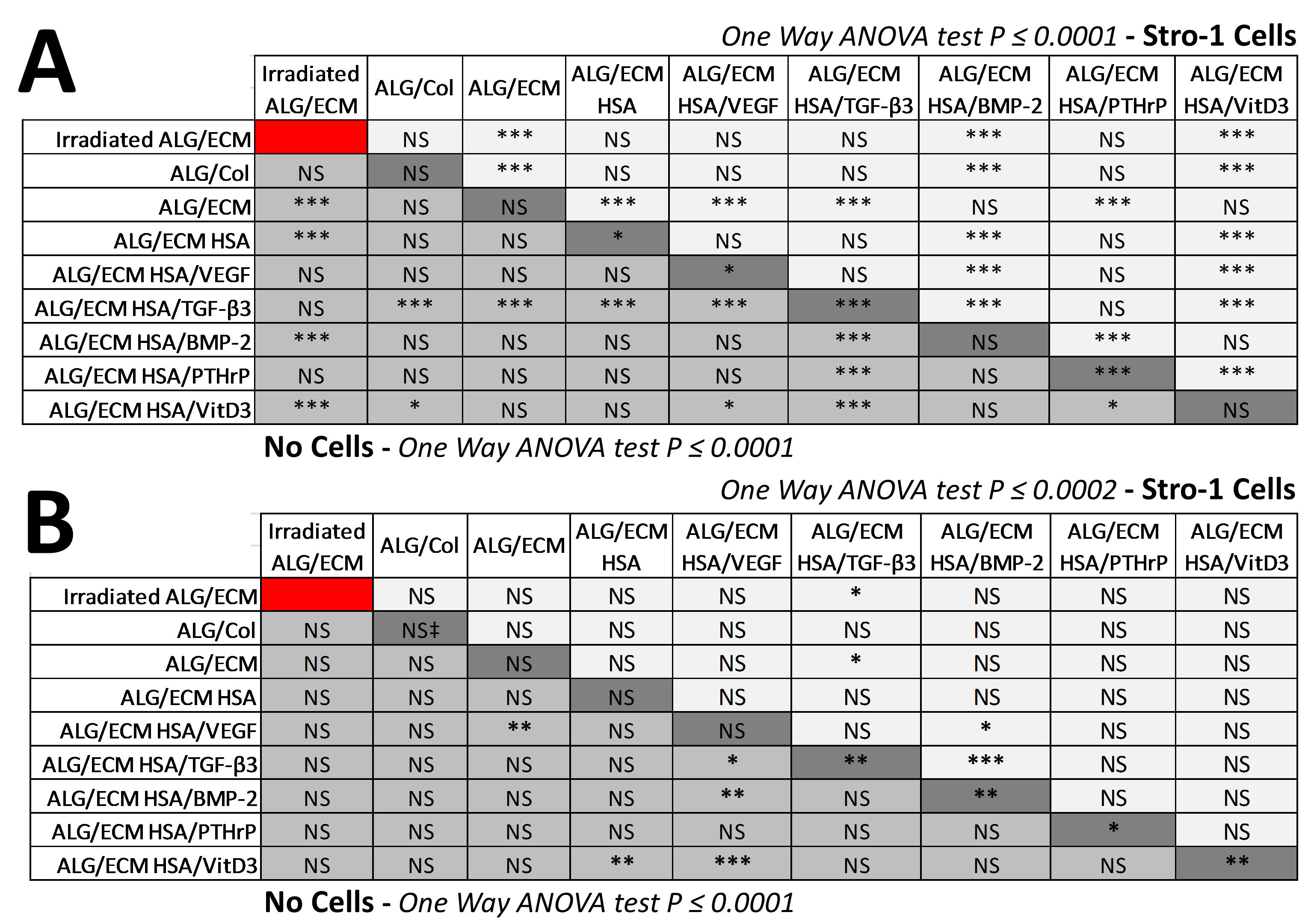

Supplement: S10 Fig — Mineralisation (A) and cell invasion (B) were both statistically analysed. Comparison between all groups with Stro-1+ cells (upper right corner–light grey) or without Stro-1+ cells (lower left corner–medium grey) were assessed by a One Way ANOVA with Tukeys post-hoc test. Dark grey boxes depict t-test comparisons within groups between those with and without Stro-1+ cells. Red box indicates non-comparison as irradiated ALG/ECM did not have Stro-1+ cells incorporated. NS indicates ‘no significance’. * P ≤ 0.05, ** P ≤ 0.01, *** P ≤ 0.001. (TIF) [file pone.0145080.s010.tif]

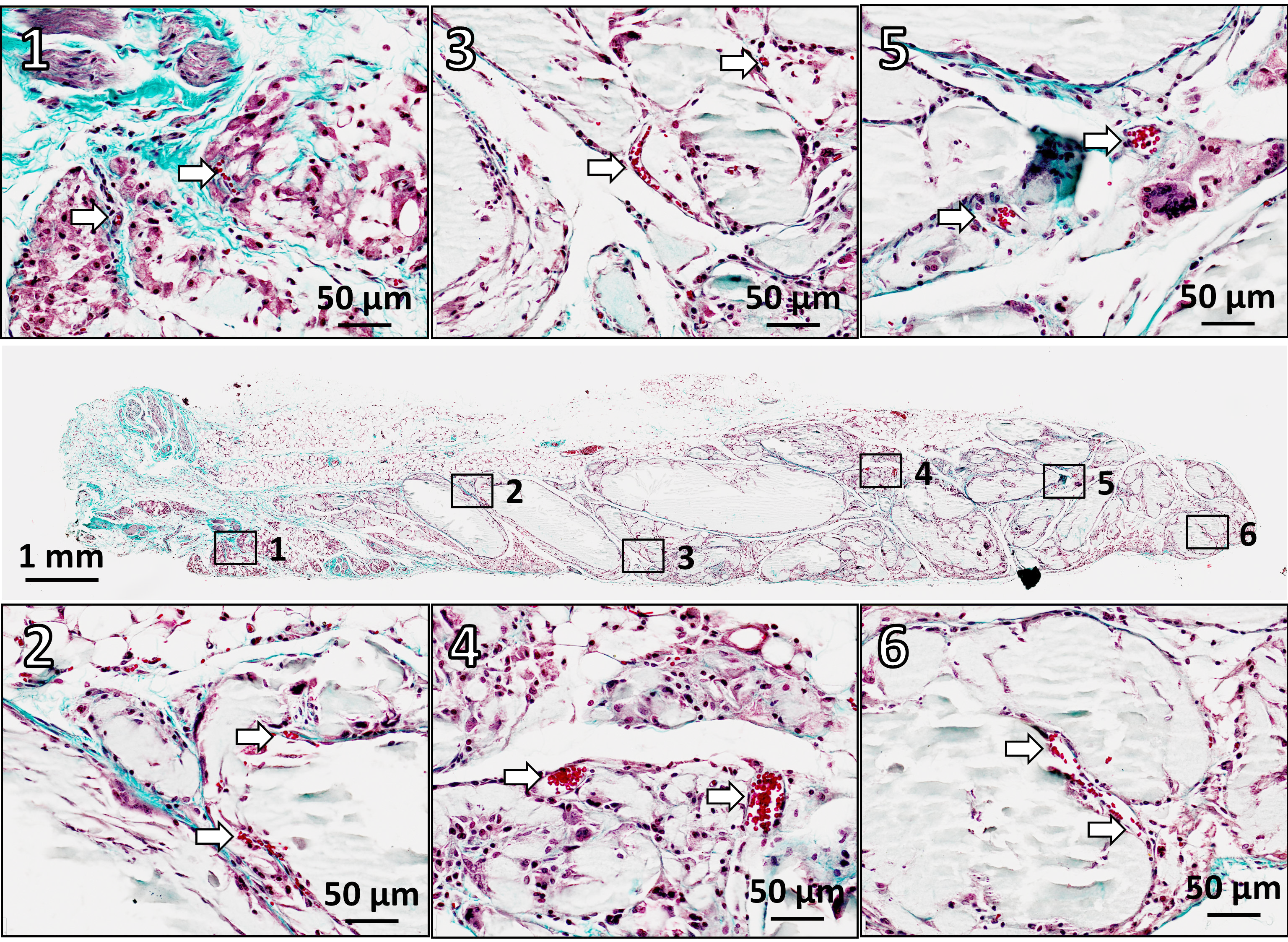

Supplement: S11 Fig — Host blood vessel invasion is depicted by white arrows within magnified areas. Image was taken from a GT stained ALG/ECM hydrogel following 28 days in vivo implantation. (TIF) [file pone.0145080.s011.tif]

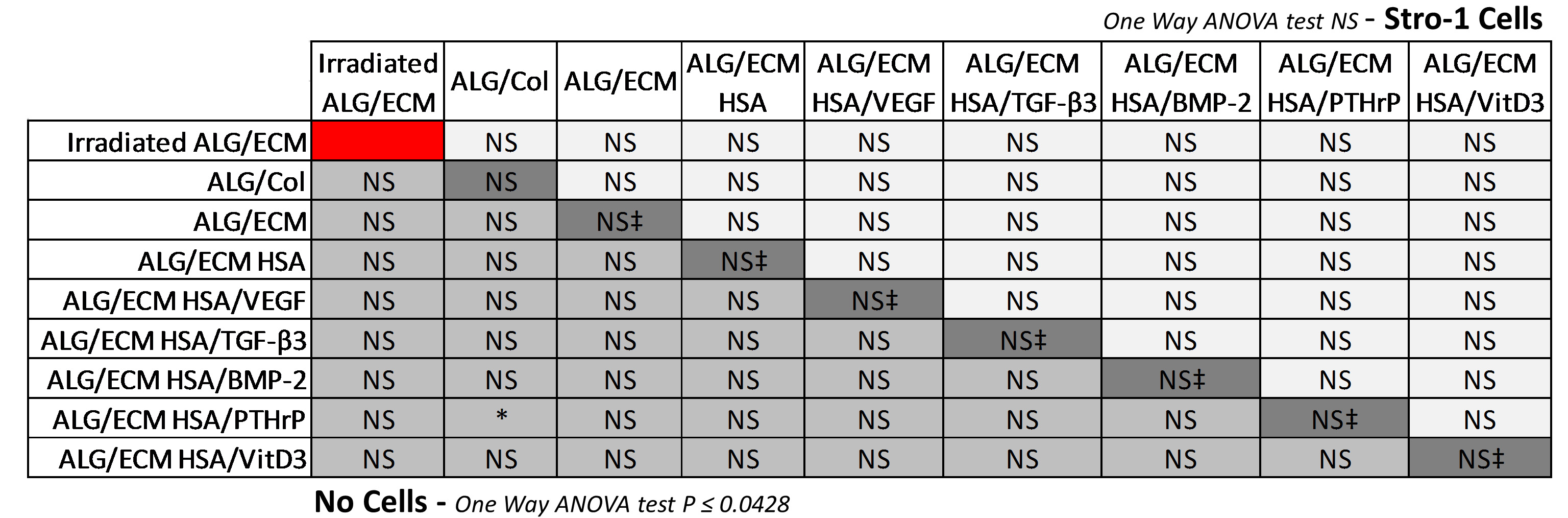

Supplement: S12 Fig — Comparisons between all groups with Stro-1+ cells (upper right corner–light grey) or without Stro-1+ cells (lower left corner–medium grey) were assessed by a One Way ANOVA with Tukeys post-hoc test. Dark grey boxes depict comparisons within groups between those with and without Stro-1+ cells. Red box indicates non-comparison as irradiated ALG/ECM did not have Stro-1+ cells incorporated. NS indicates ‘no significance’. * P ≤ 0.05. (TIF) [file pone.0145080.s012.tif]

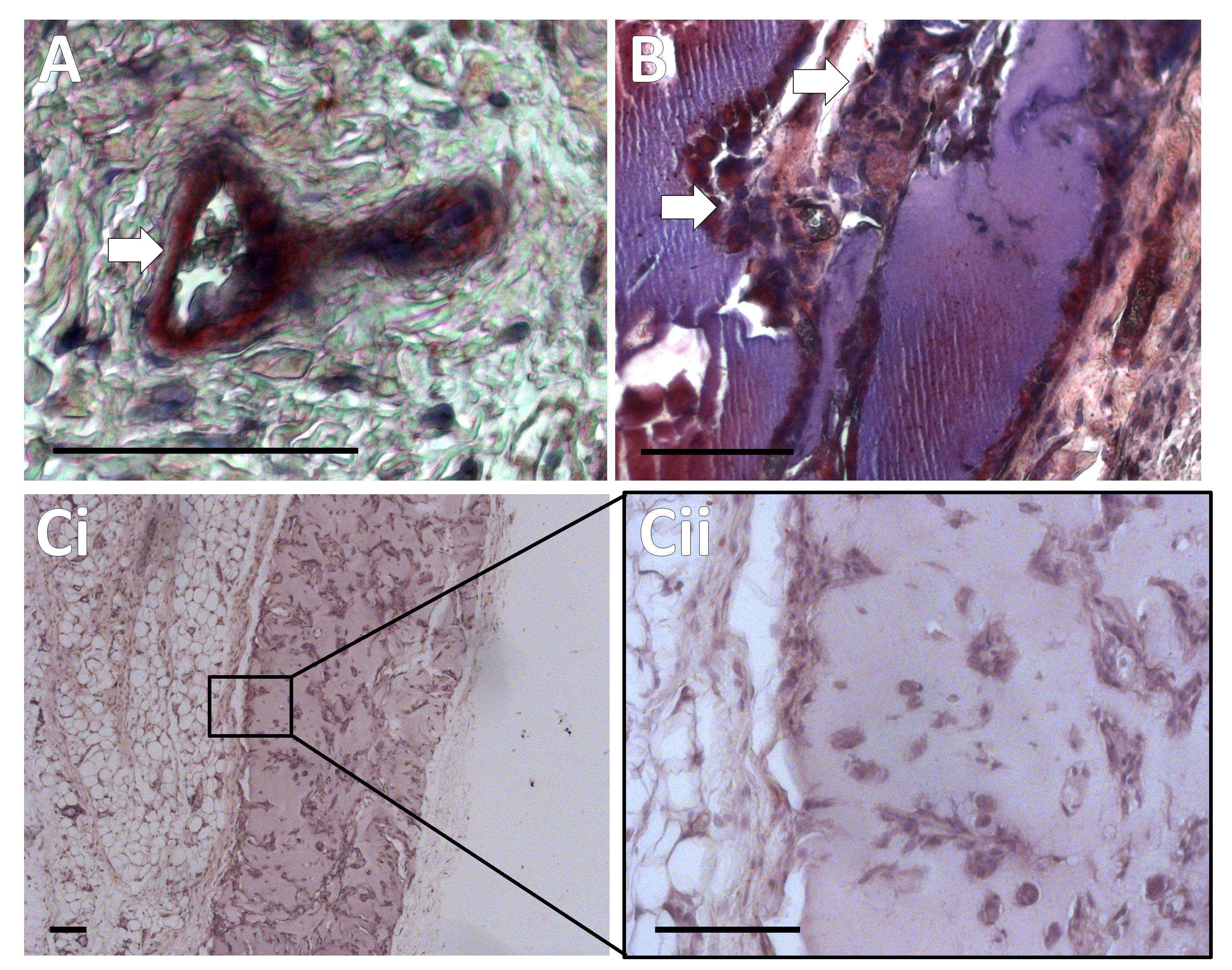

Supplement: S13 Fig — Positive red-brown staining for vWF (depicted by white arrows) identified blood vessel formation in the mouse tissue surrounding the hydrogel (A) and in the tissues invading the hydrogel (B). Samples stained for human-specific vimentin did not identify implanted Stro-1 cells (C). Sections were counterstained with haematoxylin. Scale bars measure 100 μm. (JPG) [file pone.0145080.s013.jpg]

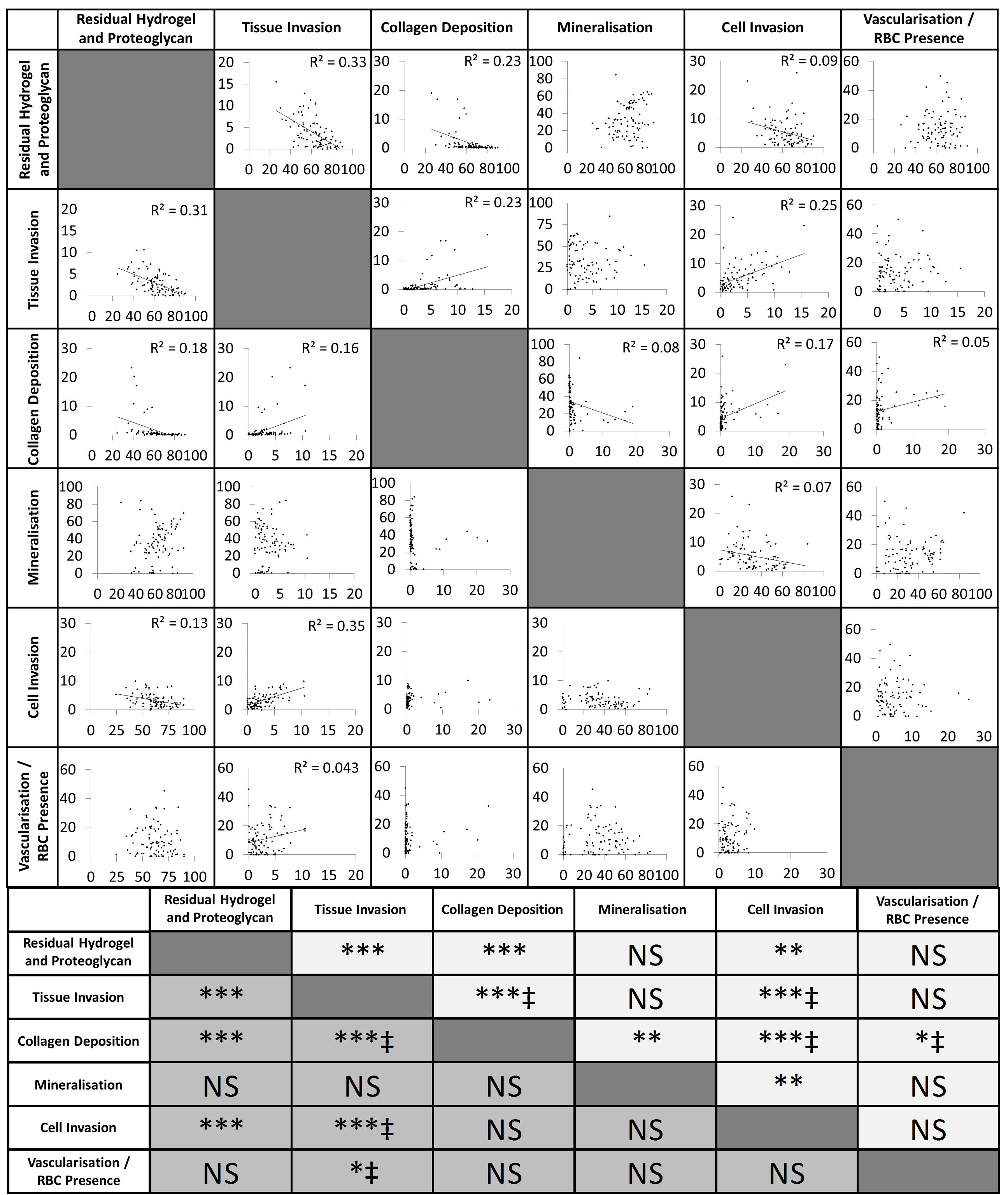

Supplement: S14 Fig — Following colour quantification of A/S, VK and GT stained samples, data for residual hydrogel and proteoglycan deposition (blue), tissue invasion (red), collagen deposition (purple), mineralisation (black), cell invasion (pink) and vascularisation (RBC presence) was combined across all growth factor groups and correlations between colours were assessed. Top right graphs depict correlations between all groups with Stro-1+ cell incorporation. Bottom left graphs depict correlations between all groups without Stro-1+ cell incorporation. Trend lines highlight those graphs depicting significant correlation. NS indicates ‘no significance’. ‡ denotes positive correlation. * P ≤ 0.05, ** P ≤ 0.01, *** P ≤ 0.001. (JPG) [file pone.0145080.s014.jpg]
